# Unexpected organellar locations of ESCRT machinery in *Giardia intestinalis* and complex evolutionary dynamics spanning the transition to parasitism in the lineage Fornicata

**DOI:** 10.1186/s12915-021-01077-2

**Published:** 2021-08-27

**Authors:** Shweta V. Pipaliya, Rui Santos, Dayana Salas-Leiva, Erina A. Balmer, Corina D. Wirdnam, Andrew J. Roger, Adrian B. Hehl, Carmen Faso, Joel B. Dacks

**Affiliations:** 1 Division of Infectious Diseases, Department of Medicine, University of Alberta, Edmonton, Alberta Canada; 2 Institute of Parasitology, University of Zurich, Zurich, Switzerland; 3 Centre for Comparative Genomics and Evolutionary Bioinformatics, Department of Biochemistry and Molecular Biology, Faculty of Medicine, Dalhousie University, Halifax, Nova Scotia Canada; 4 Institute of Cell Biology, University of Bern, Bern, Switzerland; 5 Institute of Parasitology, Biology Centre, CAS, v.v.i. Branisovska 31, 370 05 Ceske Budejovice, Czech Republic; 6 Multidisciplinary Center for Infectious Diseases, University of Bern, Bern, Switzerland; 7 Centre for Life’s Origin and Evolution, Department of Genetics, Evolution and Environment, University College of London, London, UK

**Keywords:** ESCRT, PV, *Giardia*, Evolutionary Cell Biology, Endomembrane, Parasitism, Excavata

## Abstract

**Background:**

Comparing a parasitic lineage to its free-living relatives is a powerful way to understand how that evolutionary transition to parasitism occurred. *Giardia intestinalis* (Fornicata) is a leading cause of gastrointestinal disease world-wide and is famous for its unusual complement of cellular compartments, such as having peripheral vacuoles instead of typical endosomal compartments. Endocytosis plays an important role in *Giardia*’s pathogenesis. Endosomal sorting complexes required for transport (ESCRT) are membrane-deforming proteins associated with the late endosome/multivesicular body (MVB). MVBs are ill-defined in *G. intestinalis*, and roles for identified ESCRT-related proteins are not fully understood in the context of its unique endocytic system. Furthermore, components thought to be required for full ESCRT functionality have not yet been documented in this species.

**Results:**

We used genomic and transcriptomic data from several Fornicata species to clarify the evolutionary genome streamlining observed in *Giardia,* as well as to detect any divergent orthologs of the Fornicata ESCRT subunits. We observed differences in the ESCRT machinery complement between *Giardia* strains. Microscopy-based investigations of key components of ESCRT machinery such as *Gi*VPS36 and *Gi*VPS25 link them to peripheral vacuoles, highlighting these organelles as simplified MVB equivalents. Unexpectedly, we show ESCRT components associated with the endoplasmic reticulum and, for the first time, mitosomes. Finally, we identified the rare ESCRT component CHMP7 in several fornicate representatives, including *Giardia* and show that contrary to current understanding, CHMP7 evolved from a gene fusion of VPS25 and SNF7 domains, prior to the last eukaryotic common ancestor, over 1.5 billion years ago.

**Conclusions:**

Our findings show that ESCRT machinery in *G. intestinalis* is far more varied and complete than previously thought, associates to multiple cellular locations, and presents changes in ESCRT complement which pre-date adoption of a parasitic lifestyle.

**Supplementary Information:**

The online version contains supplementary material available at 10.1186/s12915-021-01077-2.

## Background

The food- and waterborne diarrheal disease known as Giardiasis causes global healthcare and agricultural burden with approximately 300 million and more than 10 million cases diagnosed in humans and animals every year, respectively [1]. The causative agent is the diplomonad *Giardia intestinalis.* This enteric protist parasite has undergone large genome streamlining and modifications in its typical eukaryotic organelles, particularly in its endomembrane system and the associated trafficking complement [2].

*Giardia* relies heavily on its endomembrane trafficking system to secrete virulence factors while establishing gut infection, performing antigenic variation for immune system evasion, and interfering with immune responses by degrading or reducing synthesis of signalling molecules [3–7]. Endomembrane trafficking is also required for completion of the life cycle during encystation which features regulated secretion of large amounts of cyst wall material through COPII- and COPI-associated lineage-specific encystation-specific vesicles (ESVs) [8]. *Giardia*’s endomembrane organization is significantly reduced in its complexity, most notably, because it lacks a canonical Golgi apparatus, readily identifiable early and late endosomes, lysosomes, and peroxisomes [9, 10]. Simplification of the endocytic and secretory pathways in this organism is underlined by complete loss of several protein complexes associated with membrane trafficking such as AP3, AP4, AP5, TSET, and the protein complexes that are present are often reduced in their complement such as Rabs, Rab GEFs, SNAREs, and ARF GEFs [11–15]. However*, Giardia* does harbour a tubulovesicular endoplasmic reticulum (ER) thought to carry out functions of the late endosomal pathway [10]. *Giardia* also has endocytic organelles called peripheral vacuoles (PVs) which perform bulk flow uptake of nutrients from the host environment and cargo sorting for retrograde transport [16, 17].

Endosomal sorting complexes required for transport (ESCRTs) are evolutionarily ancient complexes composed of five sub-complexes, ESCRT0/Tom1, ESCRTI, II, III, and III-A and recruited onto the growing late endosomal surface in a sequential manner to induce intraluminal vesicle formation through negative membrane deformation (Additional Material 1-Supplementary Figure 1) [18]. In model eukaryotes, ESCRT machinery is required for the biogenesis of multivesicular bodies (MVBs) which have endocytic characteristics and the ability to mediate exosome biogenesis and release [19]. Additional ESCRT functions are being discovered in plasma membrane repair, autophagy functions, post-mitotic nuclear envelope scission, and others with a shared function in membrane abscission [19, 20]. This conserved protein complex is never completely lost by organisms, underlining its importance, and was already elaborated in the LECA, presumably inherited from the Asgard archaea [21–23]. Previous bioinformatics studies have shown *Giardia intestinalis *assemblage AI isolate WB (hereafter, shortened to AWB when full name of isolate is not given) to possess patchy ESCRTII, ESCRTIII, and ESCRTIIIA machinery [21]. However, key components within each of these were reported to be absent [21, 24–26].

**Fig. 1:**
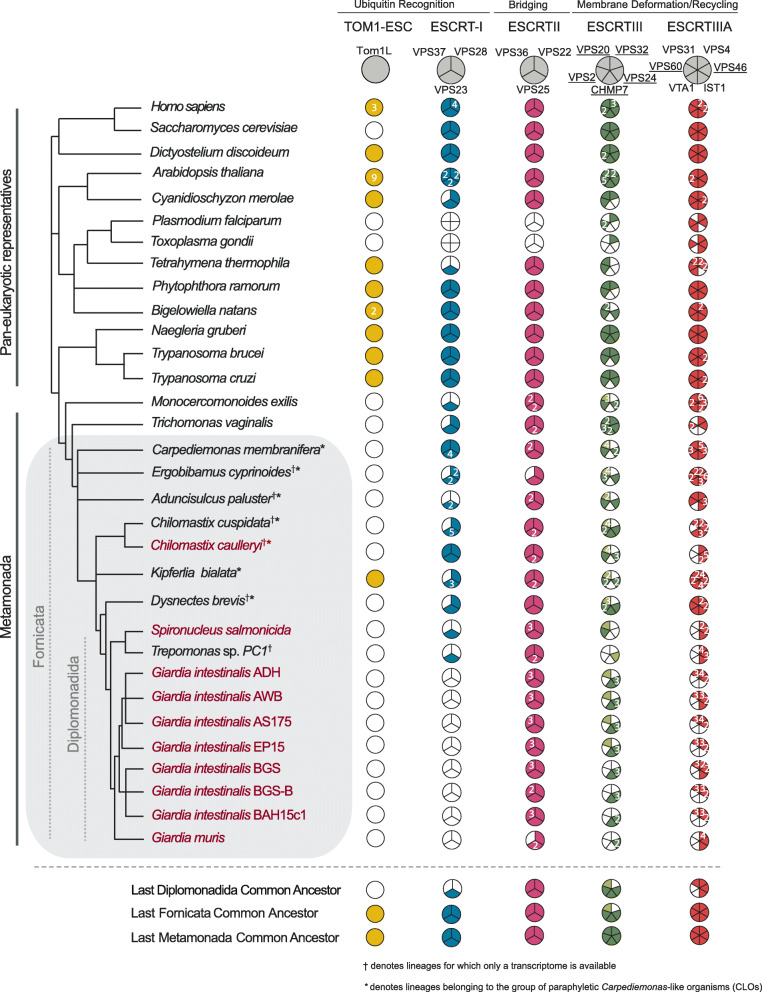
Distribution of ESCRT components within Fornicata. Coulson plot summary depicting ESCRT complement identified in Fornicata genomes and transcriptomes in comparison to pan-eukaryotic representatives. Filled sectors indicate subunits with solidified orthology determined using both comparative genomics and phylogenetics. Numbers within individual sectors represent multiple paralogues. Light coloured sectors indicate ambiguous phylogenetic classification but confirmed reciprocal blast orthology. Taxa for which genomes were available and examined are indicated in plain text whereas lineages where only transcriptomes are indicated with a superscript symbol. Lineages belonging to the paraphyletic group of *Carpediemonas*-like organisms indicated with an asterisks. Parasitic fornicates are indicated in burgundy. Paralagous ESCRTIII and ESCRTIIIA subunits possessing the SNF7 domain with common evolutionary origins, and for downstream phylogenetic investigations, are underlined. Of important note, only inferences regarding gene presence, not absences, can be made conclusively in the lineages for which only a transcriptome is available

A powerful approach to understanding the evolutionary path to parasitism is to compare protein complements in parasites with those of free-living relatives. *Carpediemonas membranifera* is a small heterotrophic flagellate, and the namesake for the paraphyletic group of free-living organisms (the *Carpediemonas*-like Organisms or CLOs) that diverged basally to the parasitic diplomonads [27]. Together, the CLOs and diplomonad parasites form the lineage Fornicata, which, in turn, are grouped with other major parasitic groups such as the parabasalids (e.g. *Trichomonas vaginalis*) or anaerobic lineages such as the Preaxostyla in the higher taxonomic ranked Metamonada (Fig. 1).

To date, although the ESCRT complement of representative metamonads (*Giardia* included) have been reported, no survey has been done of the entire Fornicata lineage nor other *Giardia intestinalis* assemblages, further raising the important evolutionary question of whether the losses reported in *Giardia* evolved concurrently with parasitism or are a product of gradual evolution that pre-date its movement into this niche.

Our initial approach using bioinformatics traces the evolution of the ESCRT system in the Fornicata, finding losses of ESCRT components across the lineage spanning the transition to parasitism. We also identified several novel components of the ESCRT machinery in *Giardia* and investigated their subcellular localization, revealing ESCRT association at PVs and other locations. Evolutionary modification of the ESCRT complement spans the transition to parasitism in the lineage leading to *Giardia*, and the modified ESCRT machinery acts at more locations than previously understood in this globally important parasite.

## Results

### ESCRT losses in Fornicata are gradual and represent a slow transition leading to parasitism

To understand the extent to which the loss of ESCRT components correlates with parasitism, versus pre-dating it, we investigated the complement encoded in the transcriptomes of free-living *Carpediemonas membranifera* and *Carpediemonas*-like organisms (CLOs) by comparative genomics (Fig. 1 and Additional Material 2-Supplementary Table 1). In the case of ESCRTIII and ESCRTIIIA SNF7 components (VPS20, VPS32, VPS60, VPS2, VPS24, and VPS46) which are themselves homologous, we also used phylogenetic analysis for classification. We took a two-step approach to account for divergent fornicate sequences, first classifying *Carpediemonas membranifera* sequences, and subsequently using these as landmarks to classify the fornicate representatives and then to verify the classification of SNF7 components.

**Fig. 2:**
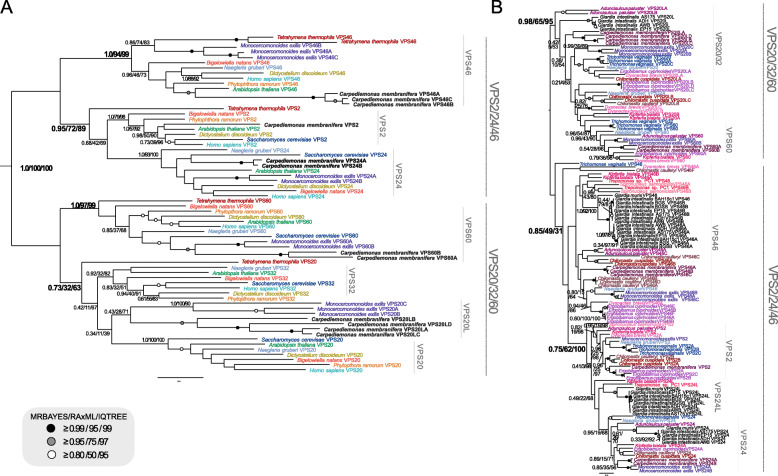
Phylogenetic analysis of ESCRTs in Fornicata. **A** Phylogenetic analyses of the ESCRTIII/IIIA SNF7 families in Fornicata. Identified ESCRTIII/IIIA SNF7 components from the basal *Carpediemonas membranifera* as a landmark representative for Fornicata were subject to phylogenetic classification. Two of the identified SNF7 sequences from *Carpediemonas membranifera* clustered clearly with VPS60 whereas the remainder neither strongly grouped with VPS20 or VPS32 and therefore were determined to be VPS20L proteins in all tree topologies. *Carpediemonas membranifera* was also determined to have VPS2, VPS24, and VP46 with strong backbone clade support for two paralogs of VPS24 (1.0/100/100) and three paralogs of VPS46 (1.0/100/100). **B** A Fornicata-specific tree with well characterized Discoba and metamonad representatives. *Monocercomonoides exilis, Trichomonas vaginalis,* and *Naegleria gruberi* as well as newly characterized sequences from *Carpediemonas membranifera* were used to classify SNF7 components in all CLOs and diplomonads. Similar to *Carpediemonas,* no clear grouping of SNF7 sequences from CLOs within the VPS20 or VPS32 clade was observed and therefore was also classified as VPS20L. Only sequences from *Giardia* AWB, ADH, and EP15 formed a group within this clade and therefore were also determined to be VPS20L. VPS2 family proteins identified in the diplomonads grouped with both VPS24 and VPS46 with duplication event pointing in *Giardia* sp. VPS46 yielding two paralogues, VPS46A and VPS46B. An additional set of SNF7 family proteins from *Giardia* AWB, ADH, and EP15 grouped with excavate and CLO VPS24 proteins therefore were determined to be VPS24-like proteins. However, an additional set of SNF7 proteins from all *Giardia* lineages formed a separate sister clade and therefore also termed to be VPS24. Trees were rooted between the VPS20/32/60 and VPS2/24/46 as previously determined by [21]

This analysis allowed us to resolve the presence of nearly all SNF7 components with clear clustering with pan-eukaryotic orthologs (Fig. 2A and Additional Material 3-Supplementary Figure 2). The exception was lack of clear VPS32 or VPS20 orthologs in *Carpediemonas.* Instead, multiple VPS20-like proteins were identified. This could imply that one of these protein paralogs may carry out the functions of canonical VPS32 or VPS20 (Fig. 2A and Additional Material 3-Supplementary Figure 2). We do not rule out the possibility that orthologs of VPS32 and VPS20 are present in the *Carpediemonas* gene repertoire which remained unexpressed in standard culturing conditions and, therefore, absent within the assembled transcriptome. Phylogenetic analyses of the identified SNF7 sequences in the remaining CLOs and diplomonads including the seven  *Giardia intestinalis* isolates further revealed that, similar to *Carpediemonas membranifera*, all VPS20 or VPS32 proteins in all Fornicata lineages have diverged to the extent that no clear clades are resolvable for either VPS20 or VPS32 (Fig. 2B and Additional Material 4-Supplementary Figure 3).

We also notably detected a CHMP7 ortholog in several fornicate representatives, including *Giardia* and the free-living *Chilomastix cuspidata* and *Dysnectes brevis* (Fig. 1). We further examined these proteins through domain analysis, which revealed that the characteristic C-terminal SNF7 domain normally required for the recruitment of downstream ESCRTIII VPS20 and VPS32 was absent from all identified CHMP7 orthologs. Following the same pattern as the VPS20L protein, this finding implies partial loss of sequence and divergence of the CHMP7 sequences pre-dates the fornicate common ancestor. Overall, our investigation of the free-living fornicate transcriptomes in direct comparison with the parasitic diplomonads and various isolates of *Giardia* has been useful in retracing the timepoints and instances of ESCRT sequence divergence.

### Losses correlating with parasitism and inter-strain variation

Focusing more specifically on parasitic lineages, including seven *Giardia* isolates, the fish parasite *Spironucleus salmonicida,* and the secondarily free-living *Trepomonas* sp. PC1, shows additional losses when compared to their free-living relatives (Fig. 1). Within the ESCRTIII machinery, we were able to classify VPS2 and VPS24 (Additional Material 5-Supplementary Figure 4). However, we were unable to classify any of the identified SNF7 proteins as canonical VPS2 proteins in either *Giardia* or *Trepomonas* sp. and instead, phylogenetic classification pointed towards homology to VPS24 and therefore these proteins were termed VPS24-like (VPS24L) proteins (Fig. 2B). Additionally, the coincident loss in all diplomonads of VTA1 and VPS60 (Fig. 1) which interact to regulate VPS4 oligomerization hints at the dispensability of the ESCRTIII-A components and that alternative factors, potential paralogs of the unidentified components, may be at play to carry out these functions [28]. Other losses common to all diplomonads include ESCRTI VPS37 and VPS28 that are not only absent in *Giardia* but also *S. salmonicida* and *Trepomonas* sp. PC1. These are indicative of adaptive genome streamlining that likely occurred in the Last Diplomonadida Common Ancestor (Fig. 1). By contrast, although greater streamlining has occurred in the diplomonads with respect to other fornicates, presence of VPS23 in *Trepomonas* sp. PC1 still hints at the capacity of these lineages to form canonical multivesicular bodies.

We also observed unanticipated protein complement differences between the two human-infecting assemblages, A and B. Assemblage A isolates, WB and DH possess two VPS24 paralogues, with one clustering with other canonical VPS24 orthologs from other excavates, the other forming a clearly separate clade, here termed VPS24L (Fig. 2B and Additional Material 6-Supplementary Figure 5). Additionally, we failed to identify any orthologs of VPS20L proteins in assemblage B isolates, GS and GS-B. Lastly, we find a similar encoded ESCRT repertoire between the assemblage A, and EP15 strains, as well as phylogenetic clustering of the EP15 sequences with ADH and AWB (Figs. 1 and 2B), consistent with a proposed closer relationship of these strains to one another than to assemblage B.

Previous work analysing only the *Giardia intestinalis* AWB ESCRT machinery reported absences in various components such as ESCRTII VPS36, ESCRTIII CHMP7, and ESCRTIIIA subunits [24, 26]. Here we show these to be present but were not previously detected probably due to high sequence divergence and the lack of the currently available genomes and or transcriptomes from free-living relatives of *Giardia* (Fig. 1 and Additional Material 2-Supplementary Table 1).

### Localization of Giardia ESCRTII VPS25 and newly identified ESCRTII VPS36 at peripheral vacuoles

Previous molecular cell biological analyses of ESCRTs in *Giardia* have been limited to highly conserved ESCRTIII and ESCRTIIIA components [25]. The bioinformatic identification of multiple newly described ESCRT components, particularly some with unclear phylogenetic affinity (e.g. VPS20L) make attractive targets for a molecular cell biological approach.

**Fig. 3:**
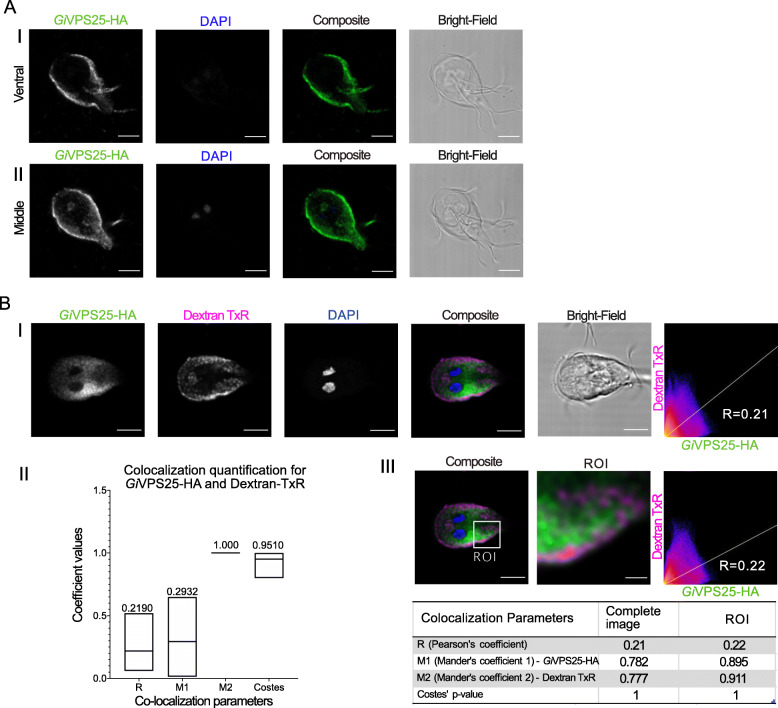
Characterization of *Gi*VPS25-HA subcellular location. **A** Trophozoite cell periphery and cytosol. (I) Ventral and (II) middle optical slice of transgenic *Giardia* trophozoite expressing epitope-tagged *Gi*VPS25-HA (green). All images were obtained using Laser Scanning Confocal Microscopy. All scale bars: 5 μm. **B** PVs. (I) Immunofluorescence assay of transgenic *Giardia* trophozoite labelled for epitope-tagged *Gi*VPS25-HA (green) and Dextran-TexasRed (magenta). (II) Distribution of co-localization parameters for *Gi*VPS25-HA and Dextran-TexasRed labeling from ≥ 15 analysed cells. Mean values for each parameter are indicated. (III) Signal overlap analysis and co-localization coefficients calculated for all slices of the sample either for the whole cell or ROI. Scale bars: composite 5 μm and ROI 1 μm. All images were obtained using Laser Scanning Confocal Microscopy


We began by characterizing the ESCRTII component VPS25, which had been consistently identified in previous phylogenetic analyses but never investigated on a subcellular level. Given previous reports on *Giardia* on ESCRTIII components and assuming functional homology from model systems, *Gi*VPS25 was predicted to associate with the PVs [25]. Immunofluorescence assays of standalone staining in transgenic trophozoites expressing *Gi*VPS25 C-terminally HA epitope-tagged reporters (*Gi*VPS25-HA), revealed an accumulation in the cell periphery and a punctate cytosolic pattern (Fig. 3A, Additional Material 7-Supplementary Video 1, and Additional Material 8-Supplementary Figure 6- Panel I). Signal overlap analyses were performed on cells (N ≥ 15) labelled for GiVPS25-HA and incubated with the endocytic fluorescent fluid phase marker Dextran coupled to Texas Red (Dextran-TxR) (Fig. 3B, Additional Material 9-Supplementary Video 2, Additional Material 10-Supplementary Figure 7- Panel I). These analyses support partial association of GiVPS25-HA to PVs (Fig. 3B—Panels II and III).

**Additional file 7: Additional Material 7-Supplementary Video 1.** Video reconstruction of *Gi*VPS25-HA confocal imaging analyses and 3-dimensional rendering of subcellular localization, as depicted in Fig. 3A.



**Additional file 9: Additional Material 9-Supplementary Video 2.** Video reconstruction of *Gi*VPS25-HA confocal imaging analyses and 3-dimensional rendering of intracellular co-localization with Dextran-TexasRed, as depicted in Fig. 3B.


To analyse the potential signal overlap between Dextran-TxR and VPS25, we performed co-localization analysis with the ImageJ/Fiji plugin Coloc2, which evaluates co-localization between two signals based on a series of computed parameters [29, 30]. These parameters are Pearson’s coefficient which computes a relation between the overlapped signal in channels of interest, Manders’ coefficients (M1 and M2) which output the percentage of each channel overlapping with the other, and the Costes p value, which determines the obtained results being true or false, where a value between 0.95 and 1 denotes true co-localization [31–33]. Between VPS25 and Dextran signal, Pearson’s coefficient describing overall signal overlap was low, as was Manders’ coefficient 1, quantifying the degree of *Gi*VPS25-HA overlap with Dextran-TxR (Fig. 3B—Panels II and III). However, Manders’ coefficient 2, describing the degree of Dextran-TxR overlap with *Gi*VPS25-HA, was high, as was the Costes value, giving us confidence in our results. Overall, these data show that the *Gi*VPS25-HA reporter localizes to PVs, consistent with past reports of other ESCRT components functioning at this organellar system. However, *Gi*VPS25-HA is also found at other locations within the *Giardia* cell.

**Fig. 4:**
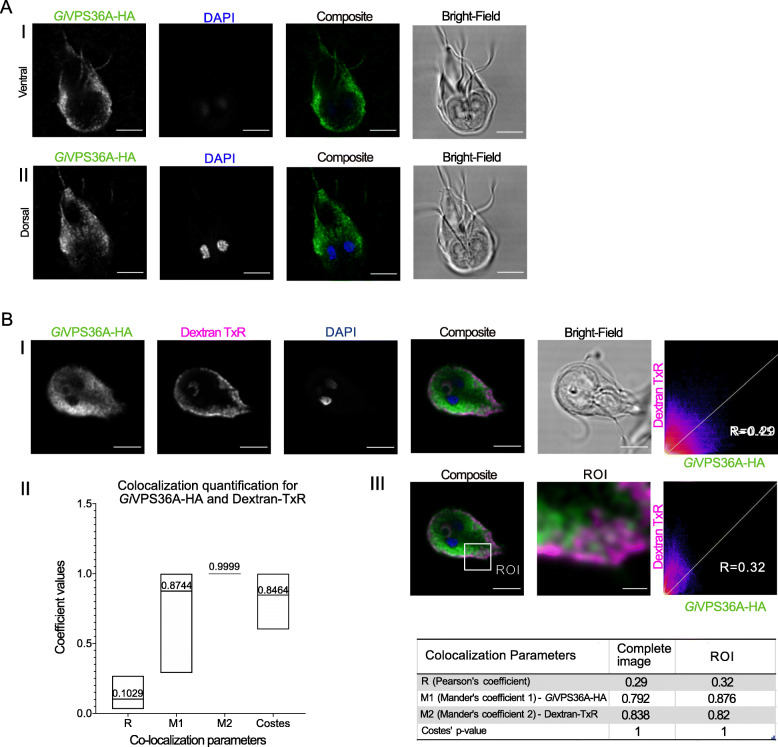
Characterization of *Gi*VPS36A-HA subcellular location. **A** Cell periphery and cytosol. (I) Ventral and (II) dorsal optical slices of transgenic *Giardia* trophozoite expressing *Gi*VPS36A-HA. All images were obtained using Laser Scanning Confocal Microscopy. All scale bars: 5 μm. **B** PVs. (I) Immunofluorescence assay of transgenic *Giardia* trophozoites labelled for *Gi*VPS36-HA (green) after incubation with Dextran-TxR (magenta). (II) Distribution of co-localization parameters for *Gi*VPS36-HA and Dextran-TexasRed labeling from ≥ 15 analysed cells. Mean values for each parameter are indicated. (III) Signal overlap analysis and co-localization coefficients calculated for all slices of the sample either for the whole cell or ROI. Scale bars: composite 5 μm and ROI 1 μm. All images were obtained using Laser Scanning Confocal Microscopy


We proceeded to characterize one of the putative ESCRT components newly identified in our bioinformatic analysis, *Gi*VPS36, hereafter referred to as *Gi*VPS36A (Additional Material 2-Supplementary Table 1). A molecular cell biological approach here is particularly informative, given that only one of the three *Gi*VPS36 paralogues possesses a potential GLUE domain, which was retrieved only weakly. In model systems, this functional module (a type of split Pleckstrin Homology (PH) domain) mediates interactions between the ESCRTI and ESCRTII sub-complexes, which are in turn necessary for ubiquitin-dependent initiation of ILV biogenesis. Instead *Giardia* VPS36 paralogues possess an N-terminal PH domain (Additional Material 2-Supplementary Table 1), raising questions of functional homology of this component with that of other model organisms [34]. We chose to test localization of *Gi*VPS36A, as this was readily identified by homology searching, and thus likely to be the least divergent in function. As with *Gi*VPS25-HA, a localization pattern associated to the cell periphery and punctate cytosolic foci was observed with detection of a *Gi*VPS36A-HA reporter construct (Fig. 4A, Additional Material 11-Supplementary Video 3, Additional Material 8-Supplementary Figure 6- Panel II). Signal overlap analyses on cells labelled for *Gi*VPS36A-HA and incubated with Dextran-TxR support partial *Gi*VPS36A-HA association to PVs (Fig. 4B—Panels II and III, Additional Material 12-Supplementary Video 4, and Additional Material 10-Supplementary Figure 7-Panel III). Manders’ coefficients again suggested PV localization, where M1 represents the signal overlap of *Gi*VPS36A-HA channel with Dextran and M2 the signal overlap between Dextran channel and the *Gi*VPS36A-HA channel. This again suggests that a very large proportion of the Dextran overlaps with VPS36 in these two channels, but that VPS36 is present where Dextran is as well as elsewhere in the cell.

**Additional file 11: Additional Material 11-Supplementary Video 3.** Video reconstruction of *Gi*VPS36-HA confocal imaging analyses and 3-dimensional rendering of intracellular localization, as depicted in Fig. 4A.



**Additional file 12: Additional Material 12-Supplementary Video 4.** Video reconstruction of *Gi*VPS36-HA confocal imaging analyses and 3-dimensional rendering of intracellular co-localization with Dextran-TexasRed, as depicted in Fig. 4B.


### Characterization of ESCRTIII VPS20L and ESCRTII components at the endoplasmic reticulum

**Fig. 5:**
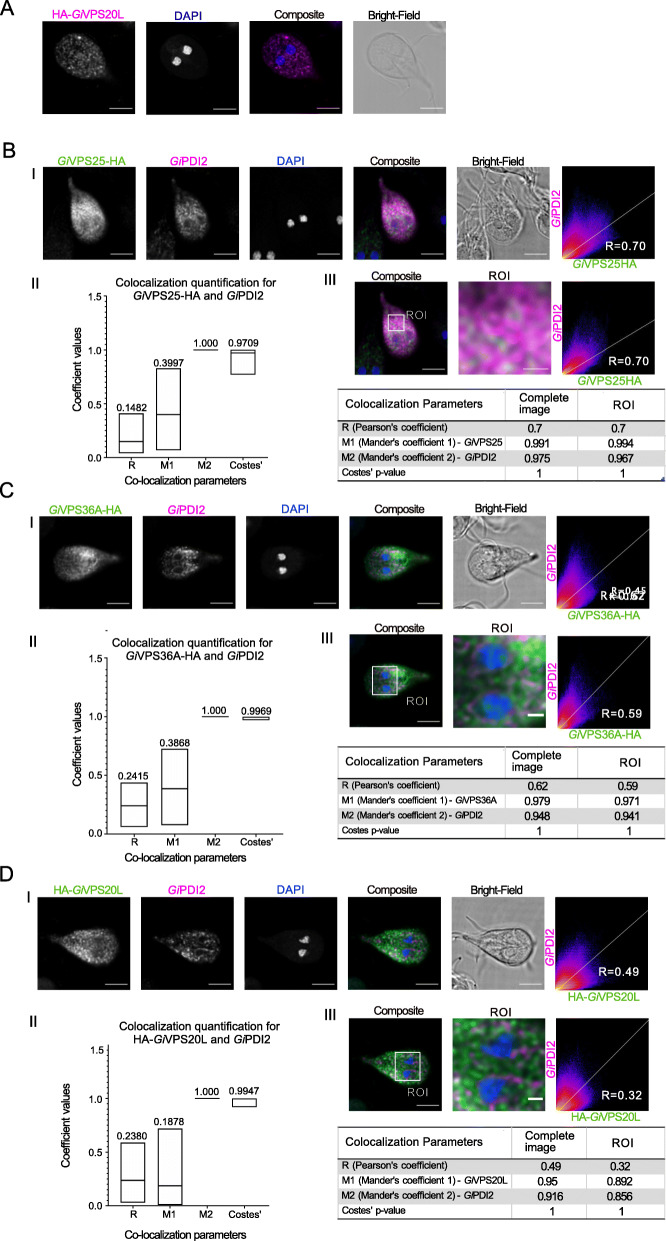
Co-labelling of *Gi*VPS25-HA, *Gi*VPS36A-HA, and *Gi*HA-VPS20L with ER membrane marker *Gi*PDI2. **A** HA-*Gi*VPS20L is found in the cytosol and punctate structures. Scale bars: 5 μm. **B–D** Panels I: Co-labelling of PDI2 (magenta) in cells expressing either **B** *Gi*VPS25-HA (green), **C** *Gi*VPS36A-HA (green), or **D** HA*-Gi*VPS20L (green). **B–D** Panels II: Mean values from ≥ 15 analysed cells for each parameter are indicated. **B–D** Panels III: Signal overlap analysis and co-localization coefficients calculated for all slices of the sample either for the whole cell or ROI. Scale bars: composite 5 μm and ROI 1 μm. All images were obtained using Laser Scanning Confocal Microscopy


The newly identified ESCRTIII VPS20L protein family was phylogenetically unresolved in our analyses and the *G. intestinalis* AWB sequence relatively divergent. Both the novelty and divergence of this protein prompted us to investigate this protein further. *Gi*VPS20L was expressed as an N-terminally epitope-tagged reporter (HA-*Gi*VPS20L) and detected by immunofluorescence localization assay (Fig. 5A, Additional Material 13-Supplementary Video 5, Additional Material 8-Supplementary Figure 6- Panel III) where we observed punctate and dispersed cytosolic localization, previously seen with *Gi*VPS25-HA and *Gi*VPS36A-HA and reminiscent of ER association of components [35].

**Additional file 13: Additional Material 13-Supplementary Video 5.** Video reconstruction of *Gi*HA-VPS20L confocal imaging analyses and 3-dimensional rendering of intracellular localization, as depicted in Fig. 5A.


This observation, along with the potential for other organellar localization suggested for VPS25 and VPS36A, prompted us to investigate whether all three proteins might be ER-associated. To do this, we proceeded with signal overlap analyses of cells (N ≥ 15) co-labelled for each epitope-tagged reporter in combination with the ER membrane marker *Gi*PDI2 (Fig. 5B–D) [8]. The data shows ESCRT proteins VPS25 (Additional Material 14-Supplementary Video 6, Additional Material 10-Supplementary Figure 7-Panel II), VPS36A (Additional Material 15-Supplementary Video 7, Additional Material 10-Supplementary Figure 7-Panel IV) and VPS20L (Additional Material 16-Supplementary Video 8, Additional Material 10-Supplementary Figure 7-Panel V) partially associate to the ER (Fig. 5B–D, Panels II and III). We interpret the low M1 coefficients (i.e. measuring the respective ESCRT components overlap with PDI) but high M2, as most consistent with the ESCRT proteins localized to ER as well as other cellular locations (e.g. the PVs).


**Additional file 14: Additional Material 14-Supplementary Video 6.** Video reconstruction of *Gi*VPS25-HA confocal imaging analyses and 3-dimensional rendering of intracellular co-localization with *Gi*PDI2, as depicted in Fig. 5B.



**Additional file 15: Additional Material 15-Supplementary Video 7.** Video reconstruction of *Gi*VPS36-HA confocal imaging analyses and 3-dimensional rendering of intracellular co-localization with *Gi*PDI2, as depicted in Fig. 5C.



**Additional file 16: Additional Material 16-Supplementary Video 8.** Video reconstruction of *Gi*HA-VPS20L confocal imaging analyses and 3-dimensional rendering of intracellular co-localization with *Gi*PDI2, as depicted in Fig. 5D.


**Fig. 6:**
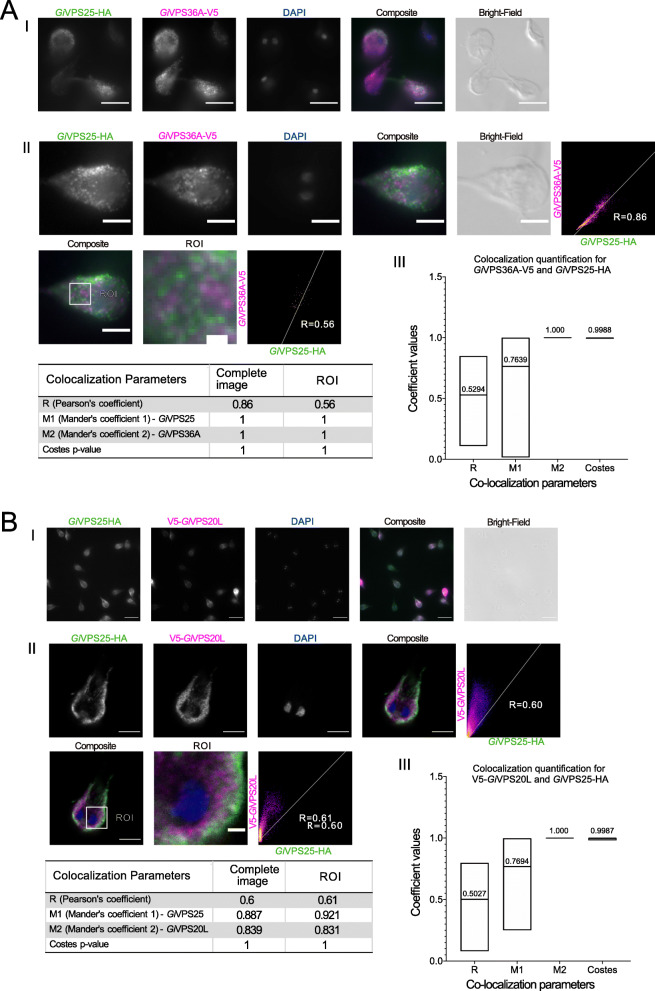
Co-expression of epitope-tagged GiVPS25 with either GiVPS20L or GiVPS36**.** Microscopy analysis of cells co-expressing *Gi*VPS25HA (green) with either **A** *Gi*VPS36A-V5 (magenta) or **B** *Gi*V5-VPS20L (magenta). Panels I: representative cell images and percentage of co-labeling. Panels II: Signal overlap analysis in both whole cells and regions of interest (ROI). Scale bars: (I) 10 μm, (II whole cell) 5 μm, and (II-ROI) 1 μm. Panels III: Mean values from ≥ 15 analysed cells for each parameter are indicated. All images were obtained using Laser Scanning Confocal Microscopy

The observation of VPS25 being in ostensibly the same locations at VPS36A and VPS20, at PVs and ER respectively, leads to the prediction of overlap in localization for these two proteins. This was assessed by developing and investigating dually transgenic *Giardia* lines expressing *Gi*VPS25-HA in combination with either *Gi*VPS36-V5 (Fig. 6A—Panel I) or V5-*Gi*VPS20L (Fig. 6B—Panel I). Based on signal overlap analysis of co-labelled cells (≥ 15), there is significant signal overlap in subcellular location for both *Gi*VPS25HA and *Gi*VPS36A-V5 (Fig. 6B—Panels II and III) and *Gi*VPS25-HA and *Gi*V5-VPS20L (Fig. 6B—Panels II and III) in co-expressing whole cells and in highlighted ROIs. Notably, Pearson’s coefficients and both Manders’ coefficients are substantially higher for ESCRT component overlap (Fig. 6) than observed for the previous co-localizations against organellar markers (Figs. 3, 4, and 5). Indeed, these values are higher for the components that in characterized model systems take part in the same sub-complex (ESCRTII), i.e. VPS25 and 36, than for VPS25 with VPS20, which is predicted to be in the ESCRTIII sub-complex. Together these all suggest a consistent picture of a multi-faceted cellular ESCRT localization in the *Giardia* cell.

### Evolutionary and protein analyses of the newly identified Giardia ESCRTIII CHMP7 reveal unsuspected ancient origins and a novel ER-Mitosomal localization

Perhaps the most surprising finding from the comparative genomics analysis was the identification of CHMP7 homologues in multiple Fornicata representatives, despite it being frequently not identified in many genomes across eukaryotes (Fig. 1). Fornicate CHMP7 proteins were also highly divergent, missing the C-terminal domain in both *Giardia* and the CLO orthologs.

**Fig. 7:**
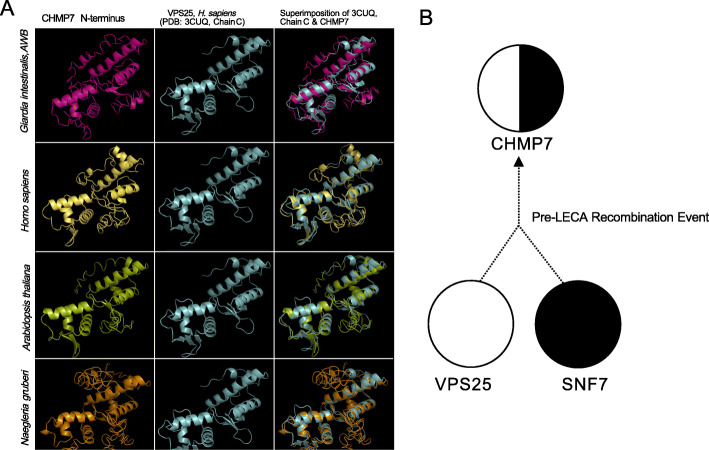
Ab initio homology-based structural analysis of the CHMP7 N-terminus. **A** Homology-based protein structural analysis of the CHMP7 N-terminus from various pan-eukaryotic representatives carried out using iTASSER ab initio structural prediction program where considerable structural similarity between the ESCRTII-VPS25 and CHMP7 N-termini. **B** Proposed evolution as determined by homology searching, structural analyses, and phylogenetic analysis (Supplementary Table S2, Supplementary Figures S8) of the pan-eukaryotic CHMP7 protein prior to the last eukaryotic common ancestor which consisted of an evolutionary fusion event between a pre-LECA ESCRTII-VPS25 and ESCRTIII/IIIA-SNF7 progenitor protein

CHMP7 is currently proposed as being derived from a pre-LECA fusion of two SNF7 domains [36]. However, potential homology of the N-terminus to VPS25 has also been proposed [37]. In order to first validate our putative CHMP7 candidates as not being divergent in-paralogs of SNF7 or VPS25, we undertook a combined phylogenetic and structural homology approach. HHPRED and iTASSER analyses of the *Giardia* CHMP7 showed a lack of a predicted C-terminal SNF7 domain. Notably, they showed sequence and structural homology of the remainder, i.e. the N-terminus of this protein, to VPS25 (Fig. 7 and Additional Material 17-Supplementary Table 2). Homology searching analyses with selected CHMP7 N-termini from several representatives of other eukaryotic supergroups confirmed this homology assessment (Fig. 7A and Additional Material 17-Supplementary Table 2) retrieving VPS25 as the only homologous protein with any significant e-values. Notably SNF7-derived proteins were never retrieved amongst the candidate homologous proteins. Given the exclusive homology to VPS25 indicated by the homology searching results, the identity of the fornicate proteins as CHMP7 and not as in-paralogues of VPS25 was also confirmed through our phylogenetic analysis (Additional Material 18-Supplementary Figure 8). Our collective structural prediction and phylogenetic findings suggest that a duplication event followed by a fusion event between the VPS20/32 SNF7 and VPS25 had occurred prior to the last eukaryotic common ancestor but subsequent to eukaryogenesis from the presumed Asgard archaeal ancestor.

**Fig. 8:**
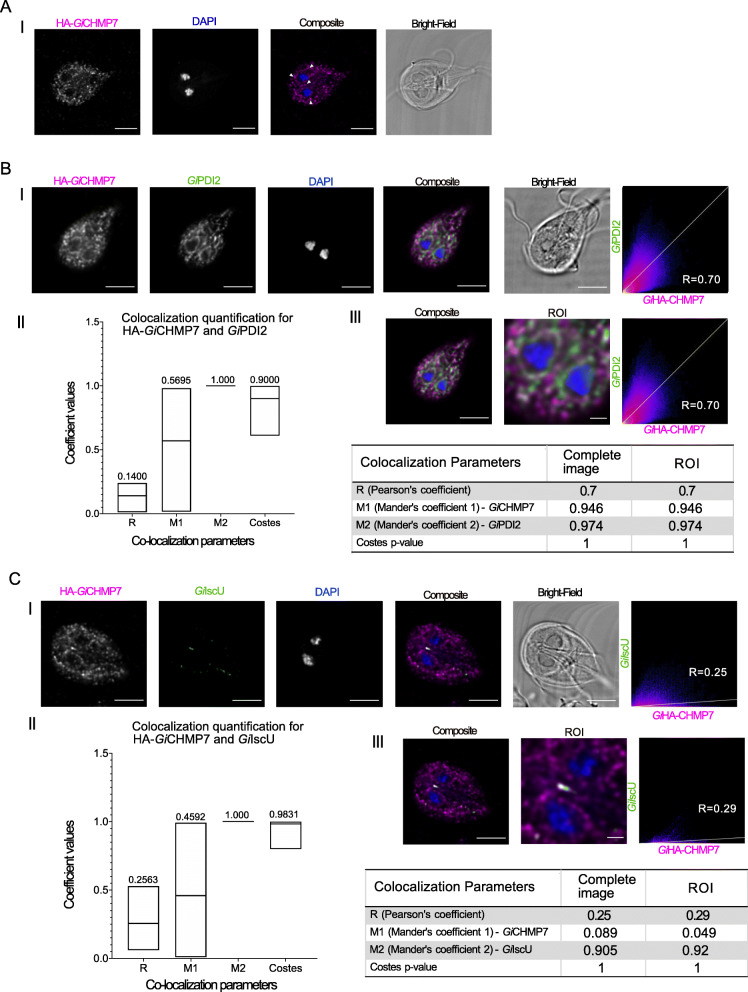
Characterization of *Gi*CHMP7 subcellular location. **A** Immunofluorescence assays of HA-*Gi*CHMP7-expressing cells yield a diffused punctate pattern with elements of perinuclear ER staining (arrowhead). Scale bars: 5 μm. **B** (I) Co-labelling of HA-*Gi*CHMP7 (magenta) -expressing cells with *Gi*PDI2 (green). (II) Distribution of co-localization parameters for HA-*Gi*CHMP7 and *Gi*PDI2 labeling from ≥ 15 analysed cells. Mean values for each parameter are indicated. (III) Signal overlap analysis for all slices of the sample either for the whole cell or ROI. **C** HA-*Gi*CHMP7 is associated to *Giardia* mitosomes. (I) Co-labelling of HA-*Gi*CHMP7 (magenta) -expressing cells with *Gi*IscU (green). (II) Distribution of co-localization parameters for HA-*Gi*CHMP7 and *Gi*IscU labeling from ≥ 15 analysed cells. Mean values for each parameter are indicated. (III) Signal overlap analysis for all slices of the sample either for the whole cell or ROI. Scale bar: composite 5 μm and ROI 1 μm. All images were obtained using Laser Scanning Confocal Microscopy


CHMP7 has been shown to have a variety of functions beyond the endocytic pathway in mammalian or yeast model cell systems [19, 38, 39]. Therefore, following the identification of this protein in *Giardia*, we aimed to investigate its role in the endomembrane system as well as its relation to the remainder of ESCRTs in this parasite. Based on *Gi*CHMP7’s similarity to VPS25 and lack of a SNF7 domain, we expected similar localization and protein interaction patterns as ESCRTII components, specifically at the PVs and at the ER. However, our immunofluorescence assay analyses with an N-terminally epitope-tagged *Gi*CHMP7 reporter (HA- *Gi*CHMP7) yielded a distinct localization pattern strongly reminiscent of ER labelling, with no obvious indication of PV association (Fig. 8A, Additional Material 8-Supplementary Figure 6 - Panel IV, and Additional Material 19-Supplementary Video 9). As done for *Gi*VPS25-HA, *Gi*VPS36A-HA and HA-*Gi*VPS20L, HA-*Gi*CHMP7 cells were co-labelled for *Gi*PDI2 and a signal overlap analysis was performed (N ≥ 15 cells), showing that HA-*Gi*CHMP7 is partially ER-associated, particularly taking M2 (i.e. signal overlap between the PDI2 channel and the ESCRT subunit channel) and the Costes values into account (Fig. 8B—Panels II and III, Additional Material 20-Supplementary Video 10, and Additional Material 10-Supplementary Figure 7-Panel VI).

**Additional file 19: Additional Material 19-Supplementary Video 9.** Video reconstruction of *Gi*HA-CHMP7 confocal imaging analyses and 3-dimensional rendering of intracellular localization, as depicted in Fig. 8A.



**Additional file 20: Additional Material 20-Supplementary Video 10.** Video reconstruction of *Gi*HA-CHMP7 confocal imaging analyses and 3-dimensional rendering of intracellular co-localization with *Gi*PDI2, as depicted in Fig. 8B.


Surprisingly, we repeatedly detected HA-*Gi*CHMP7 signal in compartments consistent with the location of central mitosome complexes (CMC) (Fig. 8C and Additional Material 21-Supplementary Video 11) [40]. To test this, we co-labelled HA-*Gi*CHMP7-expressing cells with antibodies directed against iron-sulfur cluster assembly component *Gi*IscU to detect mitosomes (Fig. 8C—Panel I, Additional Material 10-Supplementary Figure 7-Panel VII) [41]. We measured significant signal overlap limited to the CMC with *Gi*CHMP7 and *Gi*IscU-derived labels, with the low M2 denoting CHMP7 presence at multiple cellular locales, but very high M2 values indicating strong overlap with the IscU signal (Fig. 8C—Panels II and III).


**Additional file 21: Additional Material 21-Supplementary Video 11.** Video reconstruction of *Gi*HA-CHMP7 confocal imaging analyses and 3-dimensional rendering of intracellular co-localization with *Gi*IscU, as depicted in Fig. 8C.


## Discussion

*Giardia intestinalis* presents divergent cellular and genomic features and remains an enigma from an evolutionary standpoint. Our work has specifically addressed the reduced endomembrane system observed in *Giardia intestinalis*, focusing on the ESCRT protein machinery from an evolutionary and molecular cell biological perspective. We show that the reduced ESCRT complement is the product of an evolutionary process that spans the shift from free-living to a parasitic state and includes *Giardia* assemblage-specific losses. We also report on previously unidentified ESCRT machinery and unidentified sites of ESCRT location in *Giardia*, opening novel avenues for investigation.

### Gradual reductive evolution of ESCRTs and MVBs in the Fornicata

Observation of an unusual trait in a prominent parasite can lead to the default assumption that the non-canonical state is due to parasitism. But this correlation need not be causal and can be assessed by more fine-grained taxonomic sampling. Here we have assessed this exact question, regarding the ESCRT complement in *Giardia* and its parasitic and non-parasitic relatives. We have found that, while there are a few traits that do seem to correlate with the transition to parasitism in Diplomonads, the history of ESCRT system complement modulation is more textured.

**Fig. 9:**
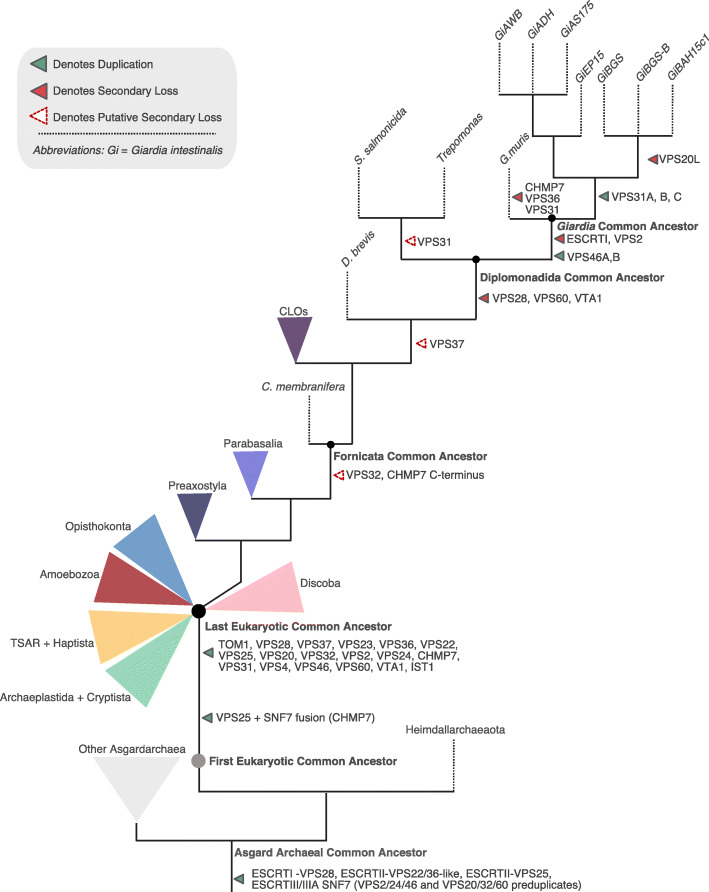
Proposed ESCRT evolution in Fornicata. Progenitor ESCRT complexes are present in Asgard archaea and duplications into the specific subunits is inferred to have occurred between the First Eukaryotic Common Ancestor and the Last Eukaryotic Common Ancestor which possessed a full complement of the ESCRT subunits. Proposed ESCRT losses in Fornicata inferred previously only using *Giardia intestinalis* are transient with some losses potentially pre-dating the Last Fornicata Common Ancestor [21, 25]. The most prominent of this being loss in CHMP7 c-terminus SNF7 domain and a canonical VPS32. Examination of diplomonad lineages, specifically genomic data, increases our confidence in additional losses also occurring with progression into parasitism most notable within the ESCRTI machinery with complete loss occurring in the *Giardia* common ancestor likely associated with a loss in the canonical MVB morphology. Speculative losses indicated as unfilled dotted arrows whereas instances of likely true gene absence depicted as solid filled arrows

Based on the lifestyles of the basally paraphyletic assemblage of CLOs, including *Carpediemonas*, the ancestor of Fornicata was likely a free-living anaerobic flagellate [42]. In these conditions, membrane trafficking machinery would be expected to play essential roles in phagotrophy, material exchange, osmoregulation, and intracellular homeostasis. From our analysis, this ancestor appears to have possessed a relatively complete complement of ESCRT machinery as compared with the deduced complement in the LECA. That said, there were likely some component losses that had already taken place (Fig. 9), including the CHMP7 SNF7 C-terminus normally required for association with the ESCRTIII VPS32. While it is technically possible that “true” orthologs of these proteins may be encoded in the not-yet sequenced genomes of CLOs, given that the pattern remains consistent across 14 different sampling points, it is much more likely for an ancestral loss to have occurred in the ancestor of fornicates, rather than multiple instances of unexpressed protein or independent losses. Loss in the SNF7 domain of CHMP7 may functionally relate to the other deduced loss observed in all free-living fornicates, that of a canonical VPS32 protein.

By contrast, the transition to parasitism appears to have happened by the time of the diplomonad common ancestor. Concurrent with this are losses of VPS28, VPS60, VTA1 and possibly VPS37 (Figs. 1 and 9). These are correlated, though not necessarily causally associated, with this transition. Notably, however, VPS23 is retained in some diplomonads and is characterized by the presence of a UEV domain which is required for interaction with cargo tagged with ubiquitin for targeted lysosomal degradation. Lineages such as *Tetrahymena*, *Entamoeba*, and *Monocercomonoides* conserving only the VPS23 from ESCRTI appear to be capable of forming functional (or at least morphologically identifiable) multivesicular bodies [43–45]. In turn, this allows us to predict that all fornicate lineages possessing ESCRTI VPS23, including the diplomonad *Trepomonas* sp. PC1, may also possess bona fide MVBs.

In the common ancestor of *Giardia* itself, we observed loss in all ubiquitin-binding components and domains. Collectively, these include TOM1-esc, ESCRTI, and VPS36 GRAM and NZF domains. We speculate that the observed lack of canonical MVB morphology in *Giardia intestinalis* specifically corresponds to losses within these components and that the existing repertoire suggests an altered role for ESCRT machinery at the *Giardia*-specific late endolysosomal organelle, the PVs. Notably, we also observed variability between the different *Giardia* genomes in some ESCRTIII and -IIIA components indicating that there is inter-strain variability in the membrane trafficking complement.

These differences are particularly evident between the two human-infecting assemblages A and B and have been noted in other membrane trafficking system proteins such as the ARF GTPase regulatory system proteins [46]. Previous reports of genome assembly and comparative genomic investigations into virulence gene families such as variant surface proteins also noted similar patterns of inter-assemblage variability. Such differences were within genome sizes (e.g. assemblage B isolates possess a genome that is approximately 11 Mb in size whereas assemblage A isolates are approximately 10 Mb), protein coding complement, and sequence-level differences in key virulence genes such as variant surface proteins, cathepsins, and cysteine proteases [47–50]. This has also lead to the postulation that in humans, Giardiasis may be caused by two different species of *Giardia* with differences in infection potential and underlying disease manifestation. Future clinical studies and *Giardia* assemblage population-level genome and comparative genomics studies should investigate this notion further.

PV vesicle-like contents have been recently observed in *Giardia* [26, 51]. However, although *Giardia* may secrete non-exosomal and non-MVB-derived extracellular vesicles, the absence of key ESCRT machinery (e.g. TOM1-esc, ESCRTI, and VPS36 GRAM domain), along with a lack of specific exosomal markers and limited proteomics data keeps the status of PV-associated vesicles in some doubt [52, 53]. An alternate interpretation is the PVs as a form of reduced and functionally limited MVB-like compartments in a similarly reduced *Giardia* endomembrane system which evolved via a process of merging organelle identity and distribution of endocytic function. Although PVs may not have a direct organellar homologue, it is still meaningful to understand which processes have been distributed to which organelles in this re-organization.

### ESCRT promiscuity at Giardia PVs, ER, and mitosomes

Previous investigations of the *Giardia* ESCRTIIIA components determined a possible role for this complex at the endolysosomal peripheral vacuoles [24–26]. While ESCRTIIIA components VPS4 and VPS46 are universally conserved in all eukaryotes, ESCRTII is not [21]. Therefore, we aimed to investigate the role of this protein complex that is usually required for bridging an existing ESCRTI and ESCRTIII in the multivesicular body pathway and how *Giardia* may be utilizing it in the absence of ESCRTI.

The imaging data and signal overlap analyses performed with tagged reporters for both *Gi*VPS25 and *Gi*VPS36 and fluorescent dextran as a soluble PV lumen marker support a PV association for both ESCRT components. The link between ESCRTs and the endocytic pathway and PVs is further corroborated by cross-referencing previously published co-IP datasets derived from PV-associated endocytic components. This highlights the presence of ESCRT proteins in these PV-centric interactomes [16, 17]. Tagged reporters for α and β subunits of AP2 collectively immunoprecipitated ESCRT components *Gi*VPS36B, *Gi*VPS36A, *Gi*VPS4A, *Gi*VPS4B, *Gi*IST1, *Gi*VPS24A, and the three *Gi*VPS31 paralogs [16]. *Giardia*’s first characterized dynamin-related protein (GiDRP) pulled down ESCRTIIIA VPS46B, VPS31A, and VPS31C. *Giardia* Clathrin heavy and putative light chains’ interactomes, similar to interactomes for the predicted PH-domain carrying PV-associated *Gi*NECAP1 protein include ESCRTIIIA subfamily components *Gi*VPS4A, *Gi*VPS4B, and the three paralogs of *Gi*VPS31 [16, 17]. This wealth of previously reported targeted proteomics data points to a clear association of *Giardia* ESCRT components to PVs, further strengthening these organelles’ status as functionally reduced and non-motile endolysosomal compartments. A clear association between ESCRT components and the ER also emerged from our investigations and is in line with reports for ESCRTIII participation in budding vesicles from the ER and for CHMP7 deposition at the perinuclear envelope in model animal/fungal systems [54, 55]. In support of our microscopy data, single co-immunoprecipitation (co-IP) experiments of epitope-tagged VPS36, VPS25, and CHMP7 each identified the transmembrane ER marker protein disulfide isomerase-2 and other PDIs as an interacting protein by mass spectrometry (Additional Material 22-Supplementary Table 3). Co-IP of epitope-tagged VPS20L did not identify any PDI proteins, but did show data consistent with interaction with Bip, another ER marker. While these data should not be taken as the basis for quantitative interactomes due to the lack of individual replicates, the fact that all four proteins co-immunoprecipitated ER markers are consistent with our conclusion of ER localization of these ESCRT components. Of note, these datasets also inform on co-immunoprecipitation of several other investigated and annotated ESCRT proteins.

The most surprising association reported here concerns the clear signal-overlap at the CMC of epitope-tagged CHMP7 and the mitosomal marker IscU, detected with confocal microscopy, from which we infer a role for this ESCRT component at mitosomes. Notably, this inference is corroborated by the presence of *Gi*CHMP7 and ESCRTIIIA components *Gi*VPS4B, *Gi*VPS46B, and *Gi*VPS31in the interactome of mitosome-localized *Gi*MOMTiP1 protein, a main interacting partner of *Gi*Tom40 [41]. Notably, the single co-IP dataset derived from epitope-tagged *Gi*CHMP7 also reciprocally detected *Gi*Tom40, albeit at low levels, by mass spectrometry (Additional Material 22-Supplementary Table 3). This is in line with the above report and our imaging data where mitosome deposition for HA-GiCHMP7 appears limited to the CMC [41]. Further data from crude sub-fractionation immunoblotting experiments using extracts of HA-GiCHMP7 transgenic cells and non-transgenic control cells also lend support to HA-GiCHMP7 association to endo-membranes (Additional Material 23-Supplementary Figure 9), consistent with the inferred presence of CHMP7 at mitosomes which contribute to these fractions, albeit also at ER [56]. Recent reports point to novel links between ESCRTs, mitochondrial membranes, and mitophagy [57–60]. Therefore, although ESCRTs have been associated to mitochondria, to our knowledge this is the first report to show an association to mitochondria-related organelles, representing a novel facet of MRO biology that should be explored in *Giardia* and in other MRO-possessing organisms.

Notably, the co-localization coefficients that we observed for the various ESCRT components told a consistent, if not entirely straightforward, story. In all cases, we observed low coefficients for overall signal overlap and degree of overlap between the ESCRT component and discrete organellar markers, but high overlap between the organellar markers and the component. The overall overlap quantification between ESCRT components, especially VPS25 and VPS20L or VPS36 however, was higher indicating that their signals were consistent. Together, this tells a story of ESCRT localization at multiple locations, beyond the PV to the ER and even the mitosome in the case of CHMP7.

### A comprehensive appreciation of ESCRT evolution and distribution in Giardia intestinalis

Definition of *Giardia* ESCRTs subcellular localizations combined with rigorous phylogenetic analyses revealed selective loss of ESCRTI which mirrors the streamlining and loss of canonical MVB morphology within Fornicata, notably in *Giardia*. In the *Giardia* lineage, we observe duplications in the ESCRTIIIA machinery with paralogs (Fig. 9) which may compensate for ESCRTI and -III losses while, in combination with remaining ESCRT components, still functioning at PVs. We further observe deep modification in *Giardia*’s ESCRT pathway by ESCRTIII components such as the CHMP7 apparently not associating within the endocytic pathway as first proposed [36].

In comparison to ESCRT machinery in characterized model organisms (Additional Material 1-Supplementary Figure 1), we observe localization of ESCRTII together with previously analysed ESCRTIIIA VPS46 and VPS4 components in close proximity to PVs and ER, while ESCRTIII CHMP7 and VPS20L seem to localize almost exclusively in regions overlapping with the ER, with additional unknown roles for ESCRTIII CHMP7 at mitosomes.

*Giardia* ESCRTIII’s association to the ER and to mitosomes presents a complex landscape of novel membrane remodelling sites while maintaining PVs as reduced and simplified MVB-like compartments mostly by the action of ESCRTII and ESCRTIIIA subunits. Our collective data sheds light on a potential mode of action for ESCRTII and ESCRTIIIA at the PV membranes. We speculate that these subunits likely associate to the PV outer membrane from a cytosolic pool and perform membrane deformation, as characteristic of other eukaryotic ESCRT subunits. Contacts sites between ER and PVs have been previously documented and could additionally be mediated by ESCRTs, allowing protein recycling down the endocytic and secretory pathway [16].

## Conclusions

We have traced the evolutionary trajectories of ESCRTs within the Fornicata, observing a slow streamlining of the ESCRT machinery across the transition to parasitism, with losses pre-dating, concurrent with, and post-dating. Several groups have recently reported on a broader set of ESCRT functions in the eukaryotic cell than previously understood. In *Giardia*, ESCRTs have been primarily previously reported at the PV. Here, we have shown ESCRT association to other membrane locations such as the ER and mitosome surface, suggesting this machinery may act more extensively at multiple organelles in *Giardia* than expected. Future functional studies should build on this comprehensive report to better assess the full range of ESCRT function.

## Methods

### Taxa studied

The previously published draft genomes of *Kipferlia bialata*, genome of *Spironucleus salmonicida*, transcriptome of *Trepomonas* sp. PC1, genome of *Giardia intestinalis* Assemblage AI, isolate WB, genome of *Giardia intestinalis* Assemblage AII, isolate DH, genome of *Giardia intestinalis* Assemblage AII, isolate AS175, draft genome of *Giardia intestinalis* Assemblage B, isolate GS, genome of *Giardia intestinalis* Assemblage B, isolate GS-B, genome of *Giardia intestinalis* Assemblage B, isolate BAH15c1 genome of *Giardia intestinalis* Assemblage E, isolate P15, and genome of *Giardia muris* were obtained from GiardiaDB and National Centre for Biotechnology Information (NCBI) [47–49, 61–67]. Latest assemblies were used in each case.

### Translation of *Carpediemonas*-like organism nucleotide transcriptomes

Nucleotide transcriptomes of *Carpediemonas membranifera* and five *Carpediemonas*-like organisms (CLOs), *Aduncisulcus paluster, Ergobibamus cyprinoides, Dysnectes brevis, Chilomastix cuspidata*, and *Chilomastix caulleryi*, were obtained from Dryad Repository and translated using the ab initio gene prediction program, GeneMarkS-T under the default parameters [42, 68].

### Comparative genomics and homology searching

Query protein sequences for individual subunits from each ESCRT sub-complex from various pan-eukaryotic representatives were obtained and aligned using MUSCLE v3. 8.31 (Additional Material 24-Supplementary Table 4) [45, 69–83]. Resulting alignments were used to generate Hidden Markov Models using the hmmbuild option available through the HMMER 3.1.b1 package and HMMER searches into all Fornicata genomes and transcriptomes using the hmmsearch tool with an e-value cutoff set to 0.01 [84]. Non-redundant forward hits were deemed positive if BLASTp reciprocally retrieved the correct ortholog in the *Homo sapiens* protein database with an e-value > 0.05 and were two orders of magnitude better in e-value than the next best hit. Reciprocal hits were extracted and sorted using an in-house Perl script.

Additional analyses of hits that failed to retrieve any reciprocal hits were analysed by BLASTp in the NCBI non-redundant database [85, 86]. Additional orthology assessment was carried out using the HHPRED suite for an HMM-HMM profile comparison and predicted secondary structure homology comparison with proteins deposited in the Protein Data Bank [87]. In order to rule out any false negatives, additional translated nucleotide (tBLASTn) searches were carried out in the Fornicata nuclear scaffolds for components that remained unidentified in HMMER searches. In cases where diplomonad sequences were unidentified due to extreme sequence divergence, identified *Carpediemonas membranifera* and CLO ESCRT orthologs were used to search the diplomonad predicted protein databases by subsequently adding these sequences into the previously generated HMM profile to build a new HMM matrix. Additionally, in order to maximize robustness of paralogue count and potential strain-specific differences, any Giardia-specific paralogues identified were then used as queries for forward BLAST searches into the other Giardia genomes. Exhaustive BLASTp and tBLASTn analyses were also performed using *Carpediemonas membranifera* and CLO sequences in the nuclear scaffolds of all diplomonads. All Fornicata ESCRT orthologs identified by this method were subject to domain analyses using Conserved Domain Database (CDD) with an e-value cutoff first set at 0.01 and then at 1.0 to detect for any highly diverged domains [88]. All confirmed hits are listed in Additional Material 2-Supplementary Table 1.

CHMP7 structural analyses was done using HHPRED as described above for selected pan-eukaryotic orthologs as well as the ab initio structural prediction tool iTASSER for protein threading and secondary structure prediction [89]. HHPRED results are summarized in Additional Material 17-Supplementary Table 2.

### Phylogenetic analyses of the ESCRTIII and ESCRTIIIA SNF7 family proteins

Phylogenetic analyses of the evolutionarily paralogous SNF7 family proteins belonging to ESCRTIII and ESCRTIIIA sub-complexes was carried out using Bayesian and maximum likelihood approaches [21]. Identified *Carpediemonas membranifera* ESCRT genes belonging to the SNF7 family were used as a landmark representative (VPS2, VPS24, VPS20, VPS32, VPS46, and VPS60) and were aligned to a pan-eukaryotic backbone containing characterized SNF7 proteins for classification into specific protein families backbone alignment containing pan-eukaryotic sequences as resolved and published by Leung et al. using the profile option in MUSCLE v3.8.31 [21, 69]. Alignments were visualized in Mesquite v3.5 and manually adjusted to remove gaps and regions lacking homology [90]. Upon classification of the *Carpediemonas* sequences, a metamonad-specific phylogenetic analysis was undertaken for the classification of identified *Giardia* and diplomonad SNF7 sequences using the same process as described above. An additional set of phylogenetic analysis was repeated using only ESCRTIII and -IIIA components, VPS2-, VPS24-, and VPS46-specific tree and VPS20-, VPS32-, and VPS60-specific tree.

Maximum likelihood approaches using non-parametric and ultrafast bootstrapping was performed using RAxML-HPC2 on XSEDE v8.2.10 and IQTREE2, respectively [91, 92]. For RAxML analyses, protein model testing was performed using ProtTest v3.4.2 [93]. In all cases, the LG + F + Γ model was used. In total, 100 non-parametric bootstraps with the default tree faster hill climbing method (-f b, -b, -N 100) were done. A consensus tree was obtained using the Consense program, available through the Phylip v3.66 package [94]. IQTREE2 best protein model selections were determined using the in-built ModelFinder package [95]. In all cases, LG + F + G4 was determined to be the best-fit model according to the Bayesian Information Criterion. Ultrafast bootstrapping with IQTREE v.2.0.6 was performed using 1000 pseudoreplicates [92]. Bayesian inference was carried out using MRBAYES v.3.0.6 on XSEDE v3.2.6 with 10 million Markov Chain Monte Carlo generations under a mixed amino acid model with number of gamma rate categories set to 4 [96]. Sampling frequency was set to occur every 1000 generations and burnin of 0.25 to discard the first 25% of samples from the cold chain. Tree convergence was ensured when average standard deviation of split frequency values fell below 0.01. A random seed value of 12345 was chosen for all phylogenetic analyses. Non-parametric and ultrafast bootstraps obtained from RAxML and IQTREE analyses were overlaid onto the MRBAYES tree topologies with posterior probabilities. RAxML and MrBAYES analyses were performed on CIPRES portal (http://www.phylo.org/index.php) while the IQTREE v.2.0.6 was installed and run locally [97]. All trees were visualized and rooted in FigTree v.1.4.4, and annotations were carried out in Adobe Illustrator CS4 [98]. All masked and trimmed alignments available upon request.

### Giardia cell culture and transfection

*Giardia intestinalis* strain AWB (clone C6; ATCC catalogue number 50803) trophozoites were grown using standard methods as described in Morf et al. [99]. Episomally transfected parasites were obtained via electroporation of the circular pPacV-Integ-based plasmid prepared in *E. coli* as previously [16]. Transfectants were selected using Puromycin (final conc. 50 μg ml^−1^; InvivoGen). Transgenic lines were generated and analysed at least thrice as soon as at least 20 million transgenic cells could be harvested, i.e. ca. 1.5 weeks post-transfection. Based on microscopy analyses of immunofluorescence assays to detect reporter levels, 85–92% of cells expressed their respective transgene(s) (Additional Material 8-Supplementary Figure 6).

### Construction of expression vectors

Oligonucleotide sequences for construct generation are listed in Additional Material 25-Supplementary Table 5 Open reading frames of interest were cloned in the pPacV-Integ vector under control of their putative endogenous promoters. Putative endogenous promoters were derived 150 bps upstream of the predicted translation start codon. ORFs were cloned in a modified PAC vector [16, 17, 100].

### Immunofluorescence assays

Chemically fixed cells for subcellular recombinant protein localization were prepared as previously described [101]. HA epitope-tagged recombinant proteins were detected using a rat-derived monoclonal anti-HA antibody (dilution 1:200, Roche) followed by a secondary anti-rat antibody coupled to AlexaFluor 594 fluorophores (dilution 1:200, Invitrogen). For co-localization experiments with ER or mitosomal markers, samples were incubated with either a mouse-derived anti-*Gi*PDI2 or a mouse-derived anti-*Gi*IscU primary antibodies both at a dilution of 1:1000, followed by incubation with anti-mouse antibodies coupled to AlexaFluor 488 fluorophores (dilution 1:200, Invitrogen) [8, 41]. For the labelling of the V5 epitope, we used anti-V5 primary antibody (1:1000; Thermo Fisher) followed by anti-mouse antibodies coupled to AlexaFluor 594 fluorophores (dilution 1:200, Invitrogen). Samples were embedded in Vectashield (VectorLabs) or Prolong Diamond Mounting medium (Invitrogen) containing 4’,6-diamidino-2-phenylindole (DAPI) for nuclear staining.

### Fluid phase marker uptake

Dextran uptake assays were performed as described in using Dextran 10 kDa TexasRed at 2 mg/mL (Invitrogen) [16, 102]. Immunostaining was performed as described above with the exception of using only 0.05% Triton-X100 (Sigma) in 2% BSA (Sigma) for permeabilization, to prevent leakage and loss of Dextran signal.

### Microscopy and image analysis

Imaging was performed using an inverted Leica SP8 Laser Scanning Confocal Microscope with appropriate parameters. Confocal images were subsequently deconvolved using Huygens Professional (https://svi.nl/Huygens-Professional) and analysed using Fiji/ImageJ [29, 30]. For co-localization analysis, the coloc2 Fiji/ImageJ plugin was used. For this, automatic background subtraction was performed in Fiji/ImageJ and 100 Costes iterations were performed [32]. Three-dimensional analysis and videos were performed in Imaris version 9.5.0 (Bitplane, AG) for Supplementary Videos 1-11. For statistical analysis of signal overlap between ESCRT subunits and specified markers, a macro was developed in Fiji/ImageJ (version 1.53d) [102]. This script has been made available through supplementary materials (Additional Material 26-Supplementary File 1). Briefly, each channel was thresholded via WEKA segmentation—a machine learning pipeline [103]. The derived binary image is used as a mask for signal overlap on ≥ 15 cells per sample/line using the Fiji plugin coloc2 [32].

### Co-immunoprecipitation with limited cross-linking

Co-immunoprecipitation assays on transgenic trophozoites expressing either HA-tagged *Gi*CHMP7, *Gi*VPS25, *Gi*VPS36A or *Gi*VPS20L, were carried out as previously shown in limited cross-linking conditions using 2.25% formaldehyde to stabilize protein complexes and enrich for weaker protein interactions during co-IP [41].

### Protein analysis and sample preparation for mass spectrometry (MS)-based protein identification

SDS-PAGE analysis was performed on 4–10% polyacrylamide gels under reducing conditions. Blotting was done as previously described by using primary rat-derived anti-HA antibody (dilution 1:500, Roche) followed by anti-rat antibody coupled to horseradish peroxidase (dilution 1:2000; Southern Biotech) [101]. Gels for mass spectroscopy (MS) analysis were stained with Instant blue (Expedeon) and de-stained with ultrapure water. MS-based protein identification was performed as previously reported [16, 17, 41, 101].

### Crude subcellular fractionation

Crude subcellular fractionation experiment was performed on HA-*Gi*CHMP7-expressing transgenic trophozoites as per previously established protocol [41]. Briefly, transgenic cells were lysed by freeze-thawing using liquid nitrogen. Soluble (supernatant) and membrane-enriched fractions (pellet) were isolated using centrifugation at 14,000×*g* for 10 min at 4 °C. Both fractions were subject to immunoprobing using a rat-derived monoclonal anti-HA antibody (dilution 1:500, Roche) followed by a secondary anti-rat antibody coupled to horseradish peroxidase (dilution 1:2000; Southern Biotech) and Coomassie staining. Non-fractionated whole cell lysates from both non-transgenic and HA-*Gi*CHMP7-expressing parasites were prepared and analysed similarly.

### In silico co-immunoprecipitation dataset analysis

The co-IP datasets derived from transgenic cells expressing epitope-tagged “baits” as affinity handles were filtered using dedicated control co-IP datasets generated from non-transgenic wild-type parasites to identify candidate interaction partners unique to bait-specific datasets [16, 17, 41]. This was done using Scaffold4 (http://www.proteomesoftware.com/products/scaffold/). Unless otherwise indicated, bait-derived co-IP data were filtered using high stringency parameters (Exclusive Spectrum Counts at 95-2-95, 0% FDR) and manually curated to rank putative interaction partners in a semi-quantitative fashion using ESCs as a proxy for relative abundance.

## Supplementary Information

**Additional file 1: Additional Material 1-Supplementary Figure 1.** The ESCRT machinery is composed of five sub-complexes each functioning consecutively for recruitment of the downstream subcomplex. The process begins with the recruitment of ESCRT0 or its analogue TOM1-esc for recognition of tagged Ubiquitin on cargo, and endosomal membrane phospholipids such as phosphatidylinositol 3-phosphate (PtdIns [3]P) upon which the ESCRTI, composed of VPS23, VPS28, and VPS37, is recruited, with its only known role being ubiquitin recognition via its UIM domain [18]. The assembly of ESCRTI then leads to assembly of the heterotetrameric ESCRTII consisting of VPS36, VPS22, and two copies of VPS25 which also bind to PtdIns [3]P via the FYVE domains [18]. Finally, this leads to the recruitment of the ESCRTIII machinery, a heteropentameric complex consisting of SNF7-domain containing family proteins, VPS20, VPS32, VPS2, VPS24, and CHMP7 [18]. A filamentous VPS32 polypeptide capped by VPS2 and VPS24 (also belonging to the paralagous SNF7-domain containing family of proteins) induces ILV formation by constricting the neck of the budding vesicle, a process which is catalysed by the ESCRTIIIA VPS4, an AAA+ ATPase [18]. It is also hypothesized that ESCRTIIIA components such as VPS31 and VPS46 are required for stabilizing the sub-complexes during the budding processes while others are needed for recycling of the complexes back into the cytosol once the process is complete [18]. Figure adapted from Stenmark and Raiborg [18].

**Additional file 2: Additional Material 2- Supplementary Table S1.** Identified ESCRT components in Fornicata genomes and transcriptomes identified and validated using HMMER, tBLASTn, BLASTP, HHPRED, and CDD domain searches.

**Additional file 3: Additional Material 3-Supplementary Figure 2.** Phylogenetic analyses of the individual VPS20-SNF7 family proteins from ESCRTIII and ESCRTIIIA sub-complexes which depicts pan-eukaryotic VPS20/32/60 with *Carpediemonas membranifera* SNF7 family proteins used as landmark representative for Fornicata. Tree inference was carried out using both BI and ML analyses. RAxML best model was determined to be LG + G + F while IQTREE ModelFinder determined an equivalent LG + G4 + F. Two of the identified SNF7 sequences from *Carpediemonas membranifera* clustered clearly with VPS60 whereas the remainder neither grouped with VPS20 or VPS32 and therefore were determined to be VPS20L proteins in all tree topologies.

**Additional file 4: Additional Material 4-Supplementary Figure 3.** Phylogenetic analyses of the individual VPS20-SNF7 family proteins belonging to the ESCRTIII and ESCRTIIIA sub-complexes depicts a Fornicata specific tree with well characterized Excavata representatives *Monocercomonoides exilis, Trichomonas vaginalis,* and *Naegleria gruberi* where no identified diplomonad SNF7 sequences grouped with VPS60. All identified *Giardia* SNF7-domain containing sequences grouped with VPS20 from other metamonads and therefore were also determined to be VPS20L sequences. Both trees were rooted at ESCRTIII-VPS60 highlighted in red [21].

**Additional file 5: Additional Material 5-Supplementary Figure 4.** Phylogenetic analyses of the individual SNF7-VPS2 family proteins from ESCRTIII and ESCRTIII-A sub-complexes depicts pan-eukaryotic VPS2/24/46 with *Carpediemonas membranifera* VPS2 family proteins used as landmark representative for Fornicata. Tree inference was carried out using both Bayesian Inference and Maximum Likelihood analyses. RAxML best model was determined to be LG + G + F while IQ-TREE ModelFinder determined an equivalent LG + G4 + F. *Carpediemonas membranifera* was determined to have all three components with strong backbone support for VPS24 (1.0/100/100) and VPS46 (1.0/100/100).

**Additional file 6: Additional Material 6-Supplementary Figure 5.** Phylogenetic analyses of the individual SNF7-VPS2 family proteins from ESCRTIII and ESCRTIII-A sub-complexes depicts a Fornicata specific tree with well characterized Excavata representatives *Monocercomonoides exilis, Trichomonas vaginalis,* and *Naegleria gruberi* in order to classify divergent diplomonad sequences. VPS2 family proteins identified in the diplomonads grouped with both VPS24 and VPS46 with duplication event pointing in *Giardia* spp. VPS46 yielding two paralogues, VPS46A and VPS46B. An additional set of VPS2 family proteins which neither grouped clearly with VPS2 or VPS24 and therefore were determined to be VPS24 like proteins. Tree was rooted at ESCRTIII-VPS46 clade [21].

**Additional file 8: Additional Material 8-Supplementary Figure 6.** Population-level expression analysis of epitope-tagged ESCRT subunits. (I) *Gi*VPS25HA is expressed in 92% of screened cells. (II) *Gi*VPS36A-HA is expressed in 86% of screened cells. (III) *Gi-*HA-VPS20L is expressed in 85% of cells while (IV) *Gi-*HA-CHMP7 is expressed in 90% of the cells. (V) Detailed results used for quantification. All scale bars: 20 μm.

**Additional file 10: Additional Material 10-Supplementary Figure 7.** Population-level analysis of cells co-labelled for ESCRT subunits and selected subcellular markers. (I) *Gi*VPS25-HA with Dextran TexasRed and (II) with *Gi*PDI2. (III) *Gi*VPS36A-HA with Dextran TexasRed and (IV) with *Gi*PDI2. (V) *Gi-*HA-VPS20L with *Gi*PDI2. (VI) *Gi-*HA-CHMP7 with *Gi*PDI2 and (VII) when counterstained for *Gi*IscU. (VIII) Detailed results used for signal overlap quantification. Scale bars: (I, V-VII) 20 μm and (II-IV) 10 μm.

**Additional file 17: Additional Material 17-Supplementary Table S2.** HHPred analyses of pan-eukaryotic, including *Giardia*, CHMP7 N-termini.

**Additional file 18: Additional Material 18-Supplementary Figure S8.** Phylogenetic analyses of the *Gi*AWBCHMP7 and *Gi*BGSCHMP7 and pan-eukaryotic and pan-eukaryotic CHMP7 N-termini against pan-eukaryotic VPS25 orthologs. HHPRED analyses (See Supplementary Table S3) of the *Giardia* CHMP7 proteins showed closest homology to ESCRTII-VPS25 and therefore were phylogenetically tested to ensure that these were in fact not additional paralogs of *Giardia* VPS25. Identified CHMP7 proteins were in fact not paralogs of the *Giardia* VPS25 which grouped in the separate VPS25 clade with the backbone support of 0.99/99/99 to the exclusion of CHMP7 clade. Tree was rooted onto ESCRTII-VPS22 pan-eukaryotic proteins.

**Additional file 22: Additional Material 22-Supplementary Table 3.** Single co-IP datasets derived from HA epitope-tagged variants for *Gi*VPS20L, 25, 36 and CHMP7 expressed in transgenic *G. intestinalis* lines.

**Additional file 23: Additional Material 23-Supplementary Figure 9.** Crude sub-cellular fractionation analysis of HA-*Gi*CHMP7. HA-*Gi*CHMP7-expressing transgenic trophozoites and non-transgenic control cells were subject to crude subcellular fractionation experiments through a freeze-thaw approach in liquid nitrogen to separate membrane-enriched and soluble fractions. Crudely fractionated protein samples, along with whole cell lysates from HA-*Gi*CHMP7 transgenic lines and WB cells as a non-transgenic negative control, were subject to blotting for immuno-detection using an anti-HA antibody, followed by a secondary anti-Rat antibody coupled to HRP. A separate Coomassie gel was also prepared and depicted to demonstrate comparable amounts of protein were loaded for detection. The full experimental approach is reported in the Methods section. M: Marker; WB: non-transgenic total lysate; WCL: whole cell lysate; F-S: crudely-fractionated sample enriched for soluble components; F-M: crudely-fractionated sample enriched for membrane components.

**Additional file 24: Additional Material 24-Supplementary Table 4.** Pan eukaryotic ESCRT queries and databases used for retrieval of up-to-date genomes and transcriptomes for homology searching and phylogenetic analyses.

**Additional file 25: Additional Material 25-Supplementary Table 5.** Oligonucleotides used for the cloning of *Giardia* ESCRT subunits of interest.

**Additional file 26: Additional Material 26-Supplementary File.** Macro for statistical analysis of labelling signal overlap between ESCRT subunits and specified markers.

## Data Availability

Pan-eukaryotic and Fornicata representative genomes and transcriptomes acquired from various databases and their corresponding GenBank assembly accessions have been summarized in Additional Material 24-Supplementary Table 4. Trimmed alignments (Supplementary Alignments 1–10) used for the phylogenetic investigations has been made publicly available through the FigShare repository (10.6084/m9.figshare.14393495.v1) [104]. Access to raw mass spectrometry data is provided through the ProteomeXchange Consortium on the PRIDE platform [105]. Data is freely available using project accession number and project DOI. Project DOI/accession number for datasets derived from bait-specific and control co-IP MS analyses are as follows: 10.6019/PXD016487 (HA-*Gi*CHMP7), 10.6019/PXD016442 (HA-*Gi*VPS20L), 10.6019/PXD016448 (*Gi*VPS25-HA), and 10.6019/PXD016446 (*Gi*VPS36A-HA) [106–109].

## Abbreviations

ESCRT: Endosomal sorting complexes required for transport
VPS: Vacuolar protein sorting
PV: Peripheral vacuoles
MVB: Multivesicular bodies
CHMP: Charged Multivesicular Protein
