# Supplementary figures and images for: Unexpected organellar locations of ESCRT machinery in Giardia intestinalis and complex evolutionary dynamics spanning the transition to parasitism in the lineage Fornicata
Source: BMC Biol. 2021 Aug 27;19:167. doi: 10.1186/s12915-021-01077-2 (PMC8394649; doi:10.1186/s12915-021-01077-2)

Supplementary Figure 1

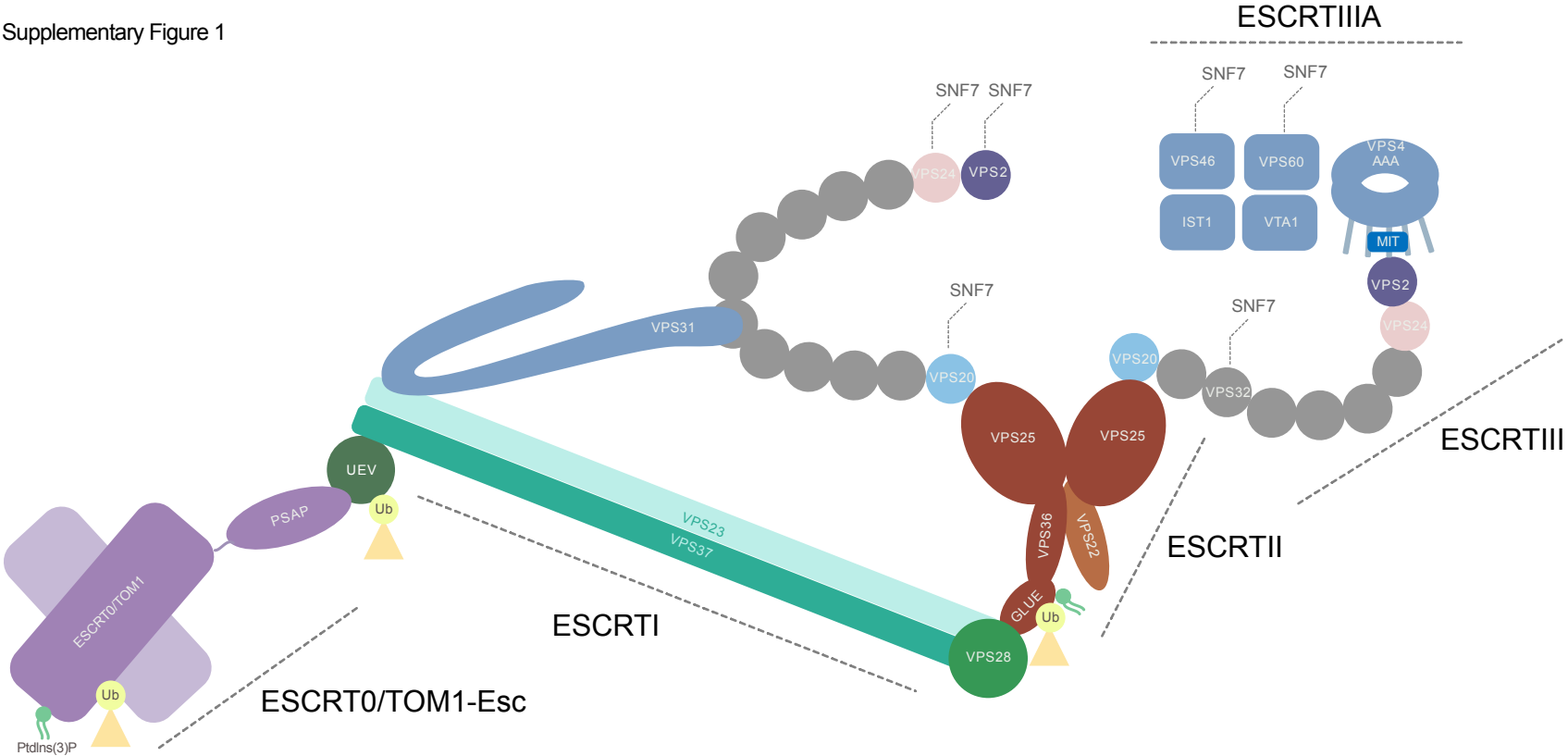

Supplement: Supplementary file 12 — Additional file 1: Additional Material 1-Supplementary Figure 1. The ESCRT machinery is composed of five sub-complexes each functioning consecutively for recruitment of the downstream subcomplex. The process begins with the recruitment of ESCRT0 or its analogue TOM1-esc for recognition of tagged Ubiquitin on cargo, and endosomal membrane phospholipids such as phosphatidylinositol 3-phosphate (PtdIns [3]P) upon which the ESCRTI, composed of VPS23, VPS28, and VPS37, is recruited, with its only known role being ubiquitin recognition via its UIM domain [18]. The assembly of ESCRTI then leads to assembly of the heterotetrameric ESCRTII consisting of VPS36, VPS22, and two copies of VPS25 which also bind to PtdIns [3]P via the FYVE domains [18]. Finally, this leads to the recruitment of the ESCRTIII machinery, a heteropentameric complex consisting of SNF7-domain containing family proteins, VPS20, VPS32, VPS2, VPS24, and CHMP7 [18]. A filamentous VPS32 polypeptide capped by VPS2 and VPS24 (also belonging to the paralagous SNF7-domain containing family of proteins) induces ILV formation by constricting the neck of the budding vesicle, a process which is catalysed by the ESCRTIIIA VPS4, an AAA+ ATPase [18]. It is also hypothesized that ESCRTIIIA components such as VPS31 and VPS46 are required for stabilizing the sub-complexes during the budding processes while others are needed for recycling of the complexes back into the cytosol once the process is complete [18]. Figure adapted from Stenmark and Raiborg [18]. [file 12915_2021_1077_MOESM1_ESM.pdf]

Supplementary Figure 2

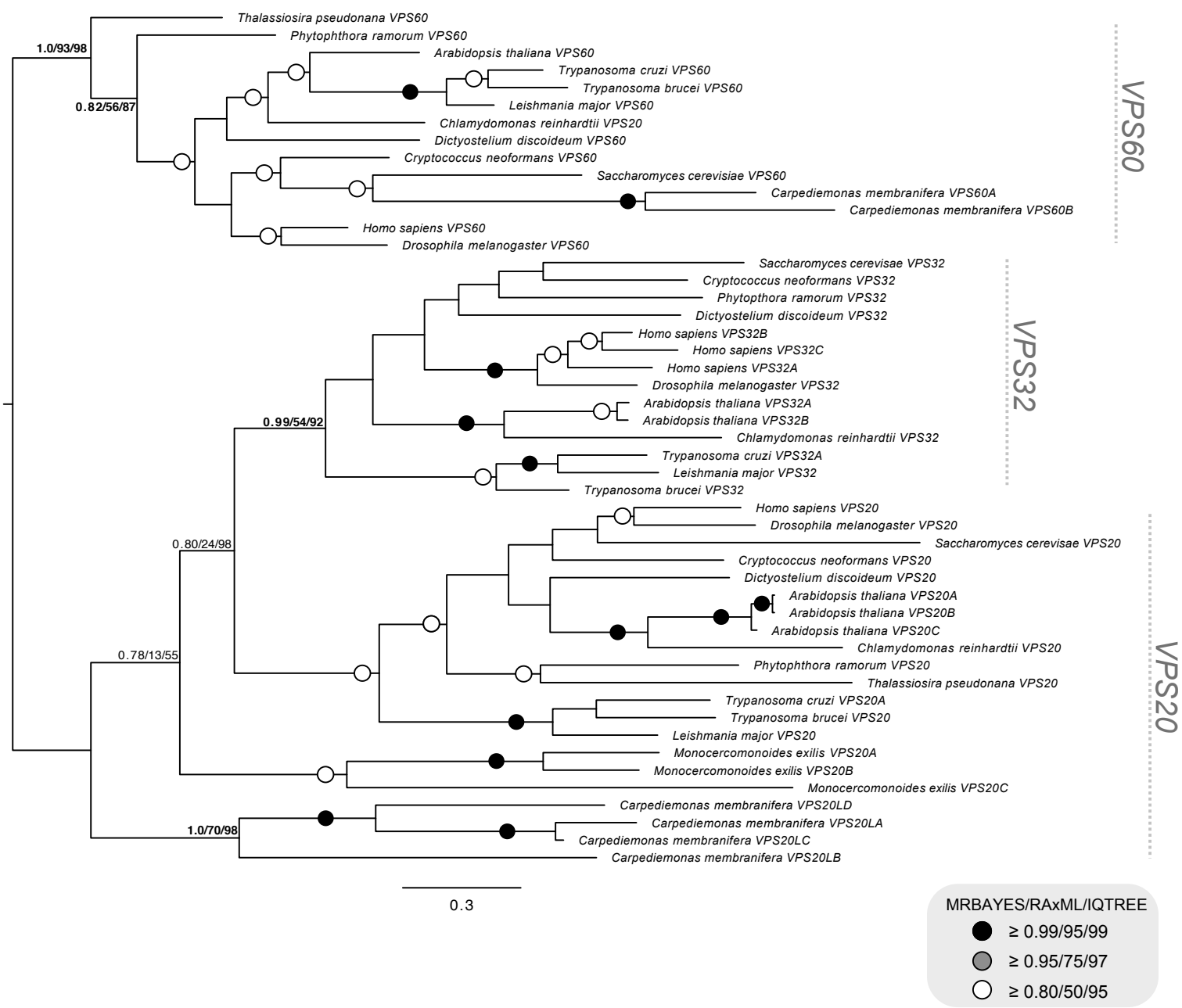

Supplement: Supplementary file 14 — Additional file 3: Additional Material 3-Supplementary Figure 2. Phylogenetic analyses of the individual VPS20-SNF7 family proteins from ESCRTIII and ESCRTIIIA sub-complexes which depicts pan-eukaryotic VPS20/32/60 with Carpediemonas membranifera SNF7 family proteins used as landmark representative for Fornicata. Tree inference was carried out using both BI and ML analyses. RAxML best model was determined to be LG + G + F while IQTREE ModelFinder determined an equivalent LG + G4 + F. Two of the identified SNF7 sequences from Carpediemonas membranifera clustered clearly with VPS60 whereas the remainder neither grouped with VPS20 or VPS32 and therefore were determined to be VPS20L proteins in all tree topologies. [file 12915_2021_1077_MOESM3_ESM.pdf]

Supplementary Figure 3

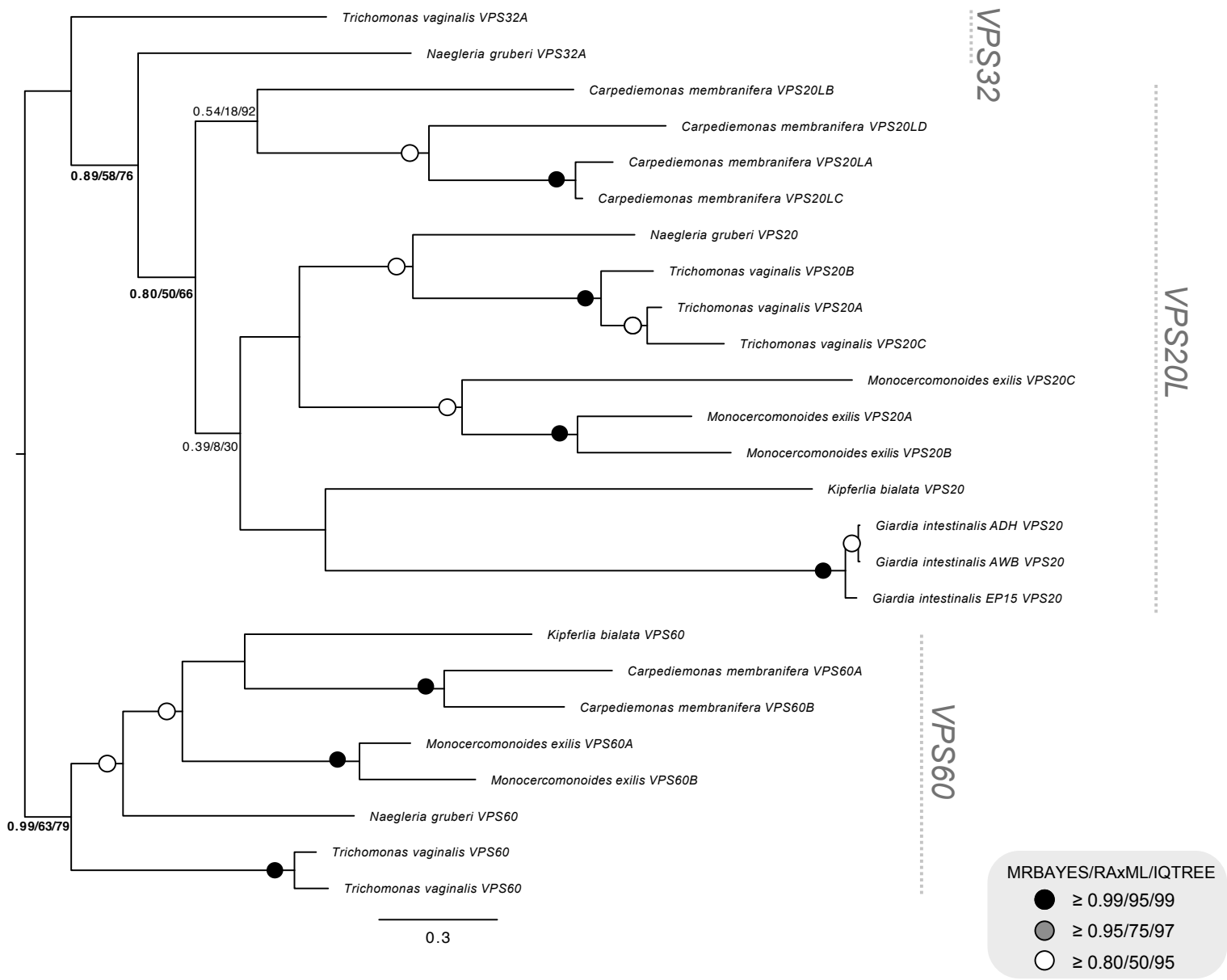

Supplement: Supplementary file 15 — Additional file 4: Additional Material 4-Supplementary Figure 3. Phylogenetic analyses of the individual VPS20-SNF7 family proteins belonging to the ESCRTIII and ESCRTIIIA sub-complexes depicts a Fornicata specific tree with well characterized Excavata representatives Monocercomonoides exilis, Trichomonas vaginalis, and Naegleria gruberi where no identified diplomonad SNF7 sequences grouped with VPS60. All identified Giardia SNF7-domain containing sequences grouped with VPS20 from other metamonads and therefore were also determined to be VPS20L sequences. Both trees were rooted at ESCRTIII-VPS60 highlighted in red [21]. [file 12915_2021_1077_MOESM4_ESM.pdf]

Supplementary Figure 4

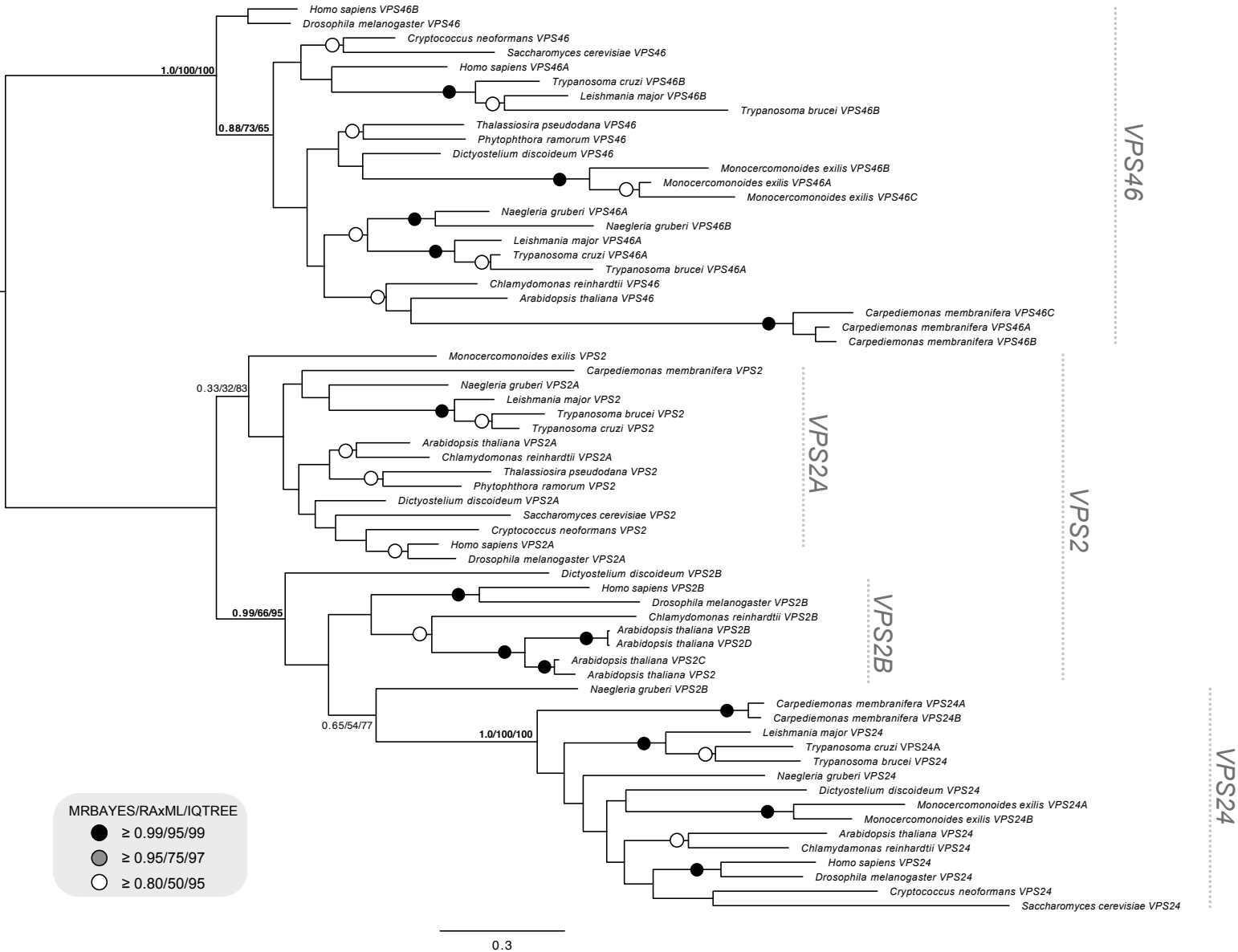

Supplement: Supplementary file 16 — Additional file 5: Additional Material 5-Supplementary Figure 4. Phylogenetic analyses of the individual SNF7-VPS2 family proteins from ESCRTIII and ESCRTIII-A sub-complexes depicts pan-eukaryotic VPS2/24/46 with Carpediemonas membranifera VPS2 family proteins used as landmark representative for Fornicata. Tree inference was carried out using both Bayesian Inference and Maximum Likelihood analyses. RAxML best model was determined to be LG + G + F while IQ-TREE ModelFinder determined an equivalent LG + G4 + F. Carpediemonas membranifera was determined to have all three components with strong backbone support for VPS24 (1.0/100/100) and VPS46 (1.0/100/100). [file 12915_2021_1077_MOESM5_ESM.pdf]

Supplementary Figure 5

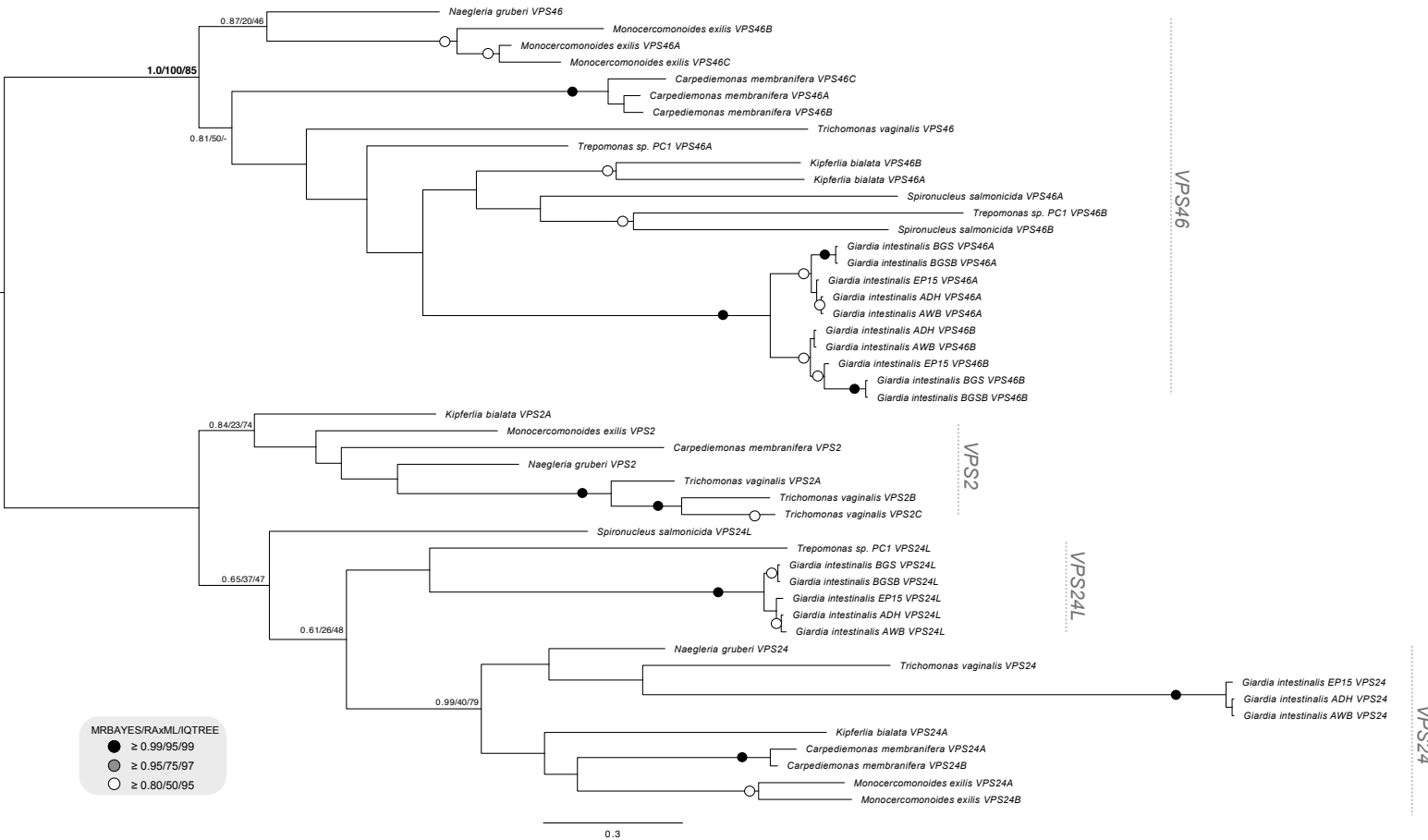

Supplement: Supplementary file 17 — Additional file 6: Additional Material 6-Supplementary Figure 5. Phylogenetic analyses of the individual SNF7-VPS2 family proteins from ESCRTIII and ESCRTIII-A sub-complexes depicts a Fornicata specific tree with well characterized Excavata representatives Monocercomonoides exilis, Trichomonas vaginalis, and Naegleria gruberi in order to classify divergent diplomonad sequences. VPS2 family proteins identified in the diplomonads grouped with both VPS24 and VPS46 with duplication event pointing in Giardia spp. VPS46 yielding two paralogues, VPS46A and VPS46B. An additional set of VPS2 family proteins which neither grouped clearly with VPS2 or VPS24 and therefore were determined to be VPS24 like proteins. Tree was rooted at ESCRTIII-VPS46 clade [21]. [file 12915_2021_1077_MOESM6_ESM.pdf]

Supplementary Figure 8

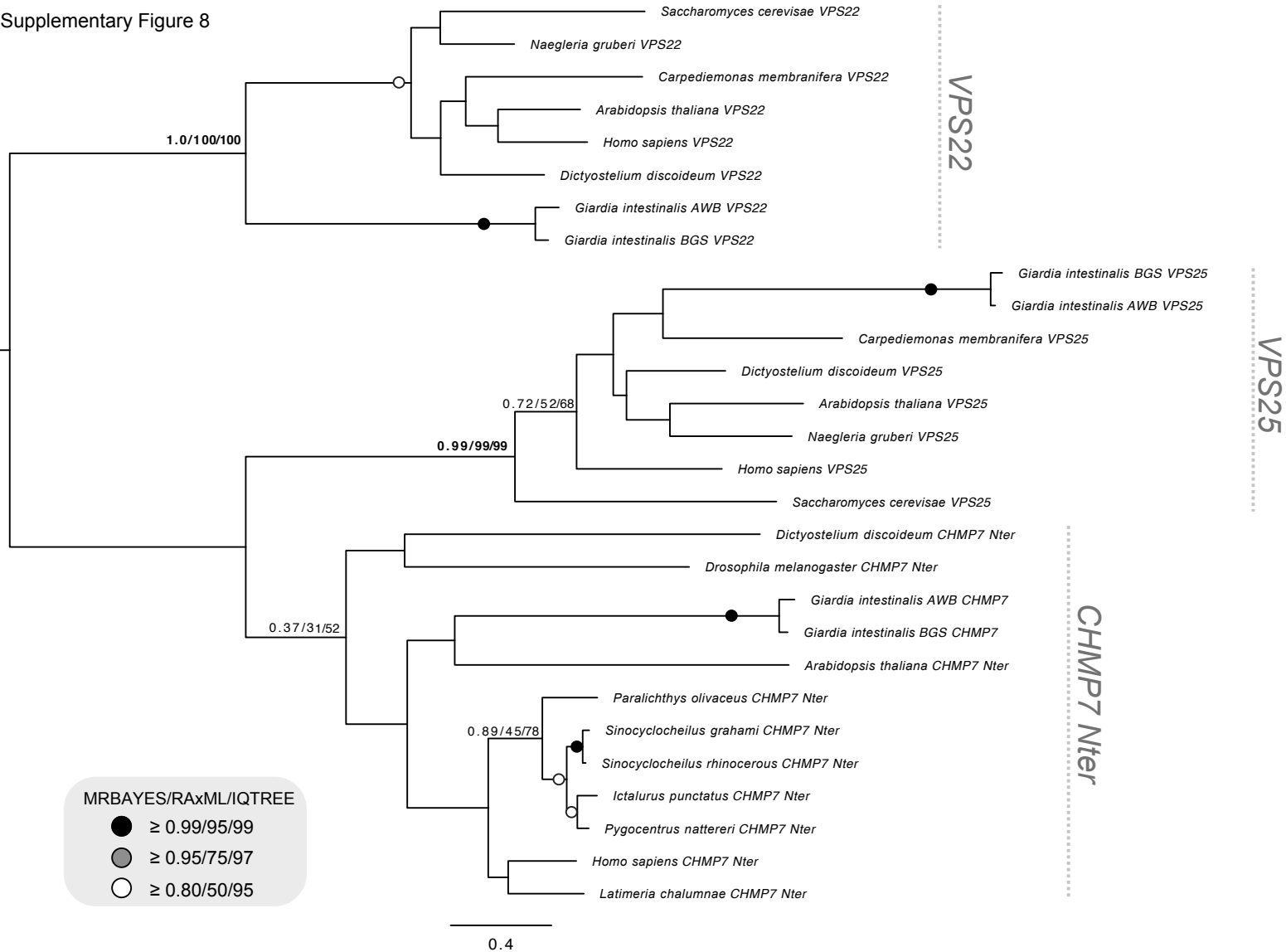

Supplement: Supplementary file 21 — Additional file 18: Additional Material 18-Supplementary Figure S8. Phylogenetic analyses of the GiAWBCHMP7 and GiBGSCHMP7 and pan-eukaryotic and pan-eukaryotic CHMP7 N-termini against pan-eukaryotic VPS25 orthologs. HHPRED analyses (See Supplementary Table S3) of the Giardia CHMP7 proteins showed closest homology to ESCRTII-VPS25 and therefore were phylogenetically tested to ensure that these were in fact not additional paralogs of Giardia VPS25. Identified CHMP7 proteins were in fact not paralogs of the Giardia VPS25 which grouped in the separate VPS25 clade with the backbone support of 0.99/99/99 to the exclusion of CHMP7 clade. Tree was rooted onto ESCRTII-VPS22 pan-eukaryotic proteins. [file 12915_2021_1077_MOESM18_ESM.pdf]
