# Supplementary material for: Unexpected organellar locations of ESCRT machinery in Giardia intestinalis and complex evolutionary dynamics spanning the transition to parasitism in the lineage Fornicata
Source: BMC Biol. 2021 Aug 27;19:167. doi: 10.1186/s12915-021-01077-2 (PMC8394649; doi:10.1186/s12915-021-01077-2)
Supplement: Supplementary file 18 — Additional file 8: Additional Material 8-Supplementary Figure 6. Population-level expression analysis of epitope-tagged ESCRT subunits. (I) GiVPS25HA is expressed in 92% of screened cells. (II) GiVPS36A-HA is expressed in 86% of screened cells. (III) Gi-HA-VPS20L is expressed in 85% of cells while (IV) Gi-HA-CHMP7 is expressed in 90% of the cells. (V) Detailed results used for quantification. All scale bars: 20 μm. [file 12915_2021_1077_MOESM8_ESM.pdf]

Supplementary Figure 6

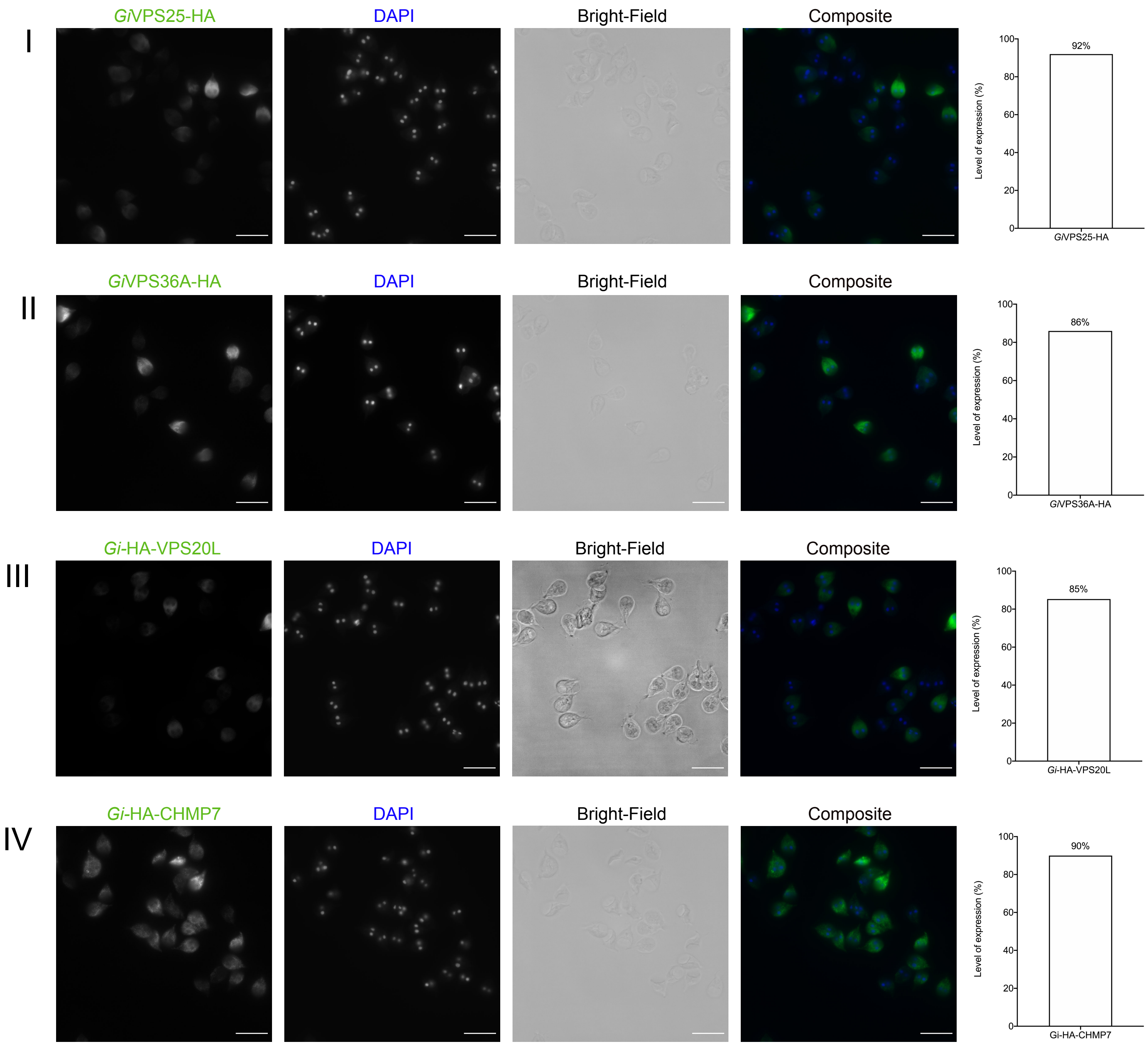

V

Cell Line	Cells Counted	Cells expressing	% of Expressing Cells
Gi VPS25HA	267	245	92%
Gi VPS36A-HA	199	171	86%
Gi -HA-VPS20L	334	285	85%
Gi -HA-CHMP7	470	423	90%
