# Supplementary material for: Unexpected organellar locations of ESCRT machinery in Giardia intestinalis and complex evolutionary dynamics spanning the transition to parasitism in the lineage Fornicata
Source: BMC Biol. 2021 Aug 27;19:167. doi: 10.1186/s12915-021-01077-2 (PMC8394649; doi:10.1186/s12915-021-01077-2)
Supplement: Supplementary file 19 — Additional file 10: Additional Material 10-Supplementary Figure 7. Population-level analysis of cells co-labelled for ESCRT subunits and selected subcellular markers. (I) GiVPS25-HA with Dextran TexasRed and (II) with GiPDI2. (III) GiVPS36A-HA with Dextran TexasRed and (IV) with GiPDI2. (V) Gi-HA-VPS20L with GiPDI2. (VI) Gi-HA-CHMP7 with GiPDI2 and (VII) when counterstained for GiIscU. (VIII) Detailed results used for signal overlap quantification. Scale bars: (I, V-VII) 20 μm and (II-IV) 10 μm. [file 12915_2021_1077_MOESM10_ESM.pdf]

Supplementary Figure 7

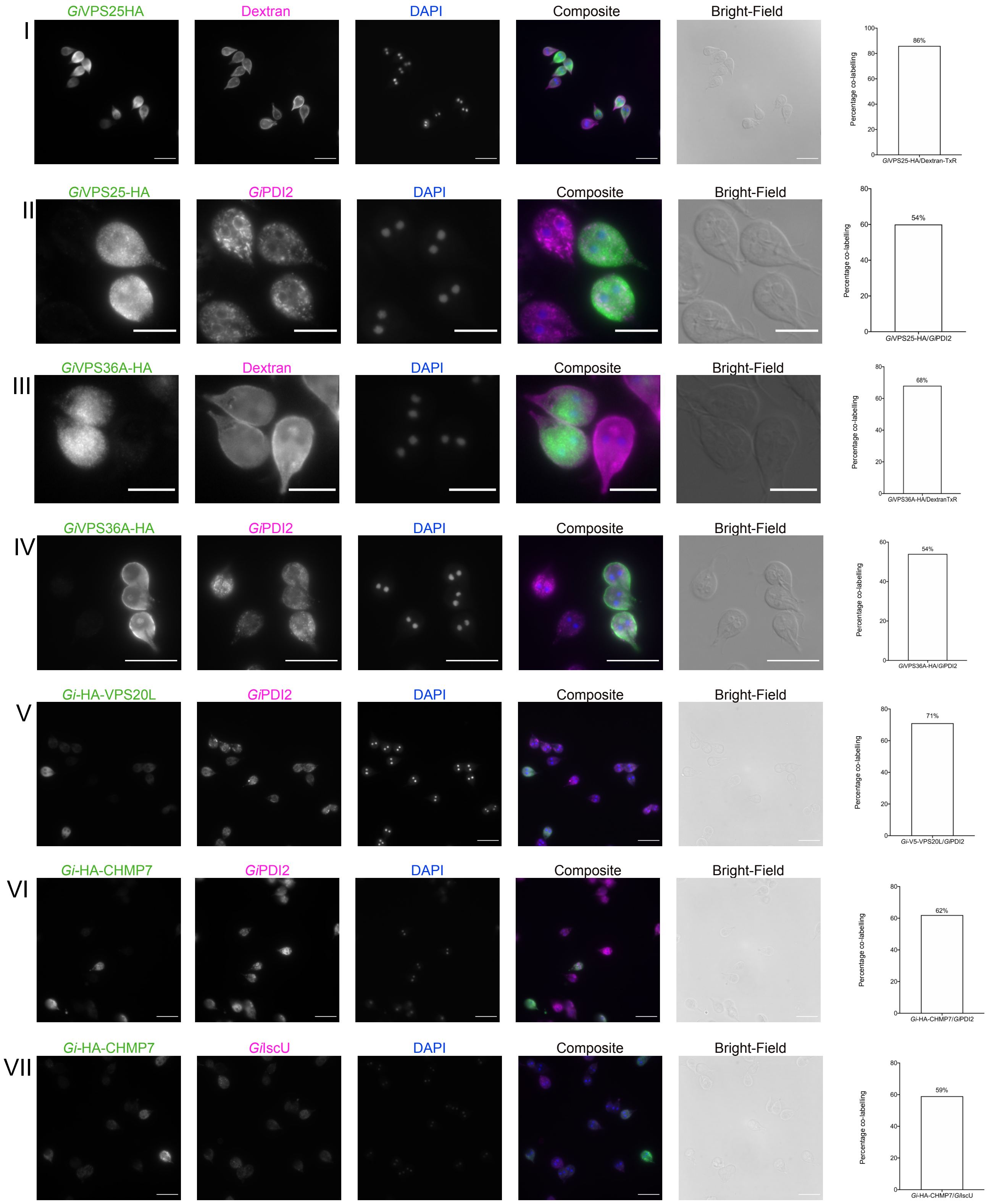

VIII

Cell Line/Condition	Cells Counted	Cells with Signal Overlap	% of Expressing Cells
Gi VPS25HA/DextranTxR	108	93	86%
Gi VPS25HA/*Gi* PDI2	53	32	60%
Gi VPS36A-HA/DextranTxR	117	79	68%
Gi VPS36A-HA/*Gi* PDI2	94	51	54%
Gi -HA-VPS20L/*Gi* PDI2	104	74	71%
Gi -HA-CHMP7/*Gi* PDI2	112	69	62%
Gi -HA-CHMP7/*Gi* IscU	102	60	59%
