# Supplementary material for: Unexpected organellar locations of ESCRT machinery in Giardia intestinalis and complex evolutionary dynamics spanning the transition to parasitism in the lineage Fornicata
Source: BMC Biol. 2021 Aug 27;19:167. doi: 10.1186/s12915-021-01077-2 (PMC8394649; doi:10.1186/s12915-021-01077-2)
Supplement: Supplementary file 23 — Additional file 23: Additional Material 23-Supplementary Figure 9. Crude sub-cellular fractionation analysis of HA-GiCHMP7. HA-GiCHMP7-expressing transgenic trophozoites and non-transgenic control cells were subject to crude subcellular fractionation experiments through a freeze-thaw approach in liquid nitrogen to separate membrane-enriched and soluble fractions. Crudely fractionated protein samples, along with whole cell lysates from HA-GiCHMP7 transgenic lines and WB cells as a non-transgenic negative control, were subject to blotting for immuno-detection using an anti-HA antibody, followed by a secondary anti-Rat antibody coupled to HRP. A separate Coomassie gel was also prepared and depicted to demonstrate comparable amounts of protein were loaded for detection. The full experimental approach is reported in the Methods section. M: Marker; WB: non-transgenic total lysate; WCL: whole cell lysate; F-S: crudely-fractionated sample enriched for soluble components; F-M: crudely-fractionated sample enriched for membrane components. [file 12915_2021_1077_MOESM23_ESM.pdf]

Supplementary Figure 9

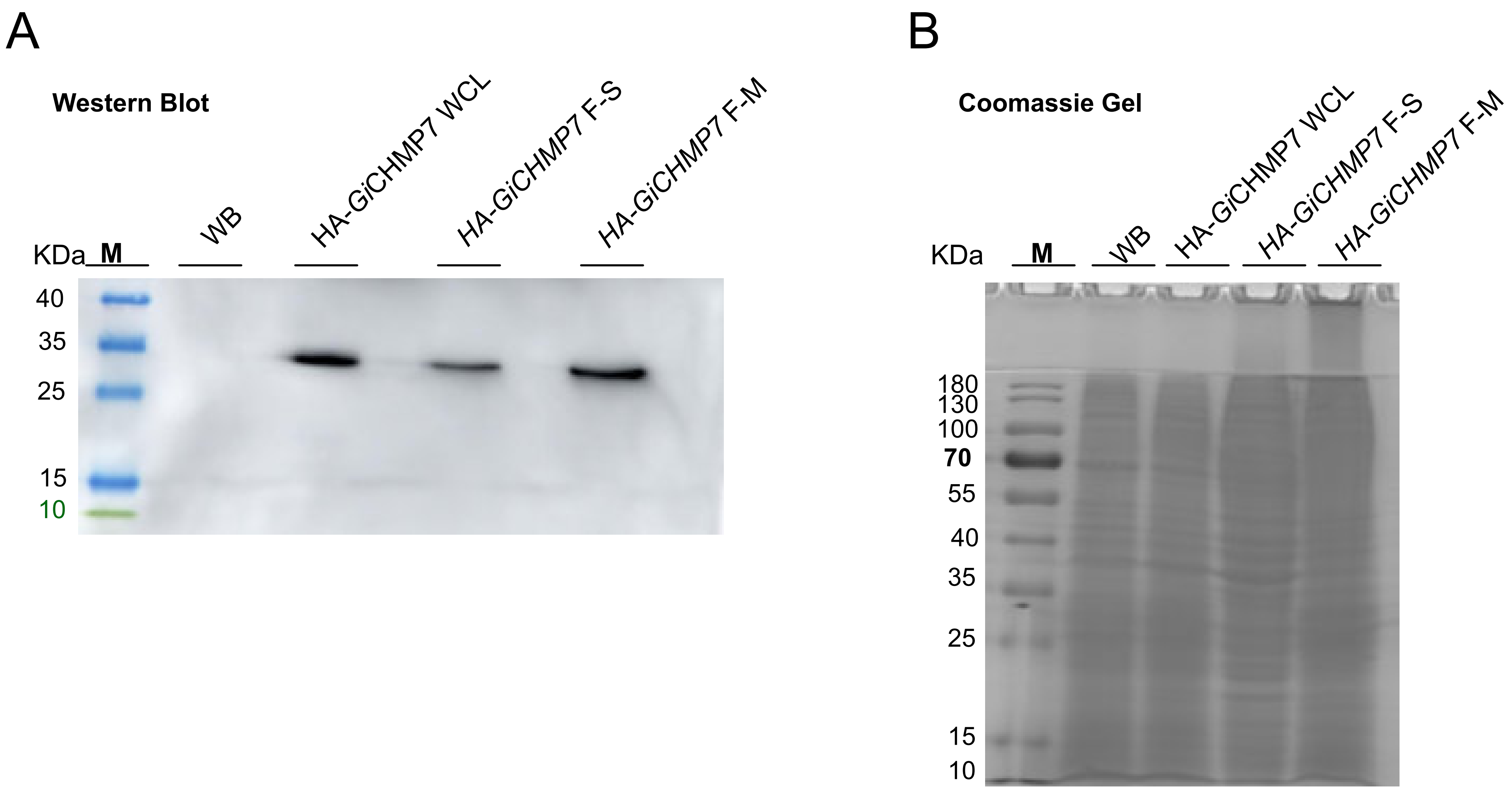

M = ThermoFischer PageRuler™ Pre-stained Protein Ladder - 10 to 180 kDa (order # 26616)

WB = non-transgenic cells

WCL = HA-*Gi*CHMP7 whole cell lysate

F-S = HA-*Gi*CHMP7 fractionated sample enriched for soluble components

F-M = HA-*Gi*CHMP7 fractionated sample enriched for membrane components
